# Altered Cardiac Autonomic Regulation in Overweight and Obese Subjects: The Role of Age-and-Gender-Adjusted Statistical Indicators of Heart Rate Variability and Cardiac Baroreflex

**DOI:** 10.3389/fphys.2020.567312

**Published:** 2021-01-28

**Authors:** Nadia Solaro, Massimo Pagani, Daniela Lucini

**Affiliations:** ^1^Department of Statistics and Quantitative Methods, University of Milano-Bicocca, Milan, Italy; ^2^Department of Medical Biotechnology and Translational Medicine, University of Milan, Milan, Italy

**Keywords:** sympathetic-parasympathetic balance, percentile rank transformation, life style, exploratory factor analysis, cardiovascular risk, non-parametric statistical inference

## Abstract

In the context of functional determinants of cardiovascular risk, a simple excess in body weight, as indexed by a rise in body mass index (BMI), plays a significant, well-recognized causal role. Conversely, BMI reductions toward normal result in an improvement of risk. Obesity is associated with impaired cardiac autonomic regulation (CAR), through either vagal or sympathetic mechanisms, which could favor the tendency to foster hypertension. Here we study the changing properties of the relationship between increasing grades of BMI and CAR in a population of 756 healthy subjects (age 35.9 ± 12.41 years, 37.4% males, 21.6% overweight, and 16% obese). Evaluation of CAR is based on autoregressive spectral analysis of short-term RR interval and systolic arterial pressure variability, from which a multitude of indices, treated overall as autonomic nervous system (ANS) proxies, is derived. Inspection of the study hypothesis that elevated BMI conditions associate significantly with alterations of CAR, independently of age and gender, is carried out using a mix of statistical transformations, exploratory factor analysis, non-parametric testing procedures, and graphical tools particularly well suited to address alterations of CAR as a disturbed process. In particular, to remove the effects of the inter-individual variability, deriving from components like age, gender or ethnicity, and to reduce the number of ANS proxies, we set up six age-and-gender-adjusted CAR indicators, corresponding to four ANS latent domains (oscillatory, amplitude, pressure, and pulse), cardiac baroreflex regulation, and autonomic nervous system index (ANSI). An impairment of the CAR indicators is overall evident in the overweight group and more marked in the obesity group. Empirical evidence is strong (9/9 concordant non-parametric test results) for pressure domain, almost strong (8/9) for ANSI, medium-strong for baroreflex (6/9) and pulse (7/9), weak for oscillatory (2/9) and amplitude (1/9) domains. In addition, the distribution of the CAR indicators corresponding to pressure, pulse, baroreflex, and ANSI is skewed toward the unfavorable abscissa extremity, particularly in the obese group. The significant association of increased BMI with progressive impairments of CAR regarding specifically the pressure domain and the overall ANS performance might underscore the strong hypertensive tendency observed in obesity.

## Introduction

Non-communicable diseases have become the primary cause of health concern worldwide, accounting for about 63% of annual deaths (Arena et al., 2015). In 2017, behavioral, environmental and occupational, and metabolic risks accounted for 34 million deaths (GBD 2017 Risk Factor Collaborators, 2018). In this context, risk factor ranks resulted: high systolic arterial pressure, smoking, high fasting plasma glucose, and high body mass index (BMI), which alone was responsible for 4.7 million deaths and 148 million DALYs (Disability-Adjusted Life Years). BMI was also among the risks that increased most in the last decade. The rise in metabolic risk might also lead to growing cardiovascular mortality, coincidentally calling for more successful risk reduction strategies (GBD 2019 Risk Factors Collaborators, 2020). It follows that our current delivery model is poorly constructed to manage chronic disease. It may be best to base our approach on patient-centered technologies (Milani et al., 2004; Lucini et al., 2020) and rely on simple lifestyle interventions such as healthy diet and physical exercise (Fock and Khoo, 2013).

Considering that obesity is associated with increases in all-cause and cardiovascular mortality (Jensen et al., 2014), it is advisable to investigate in every patient all possible concomitant risk factors (hypertension, dyslipidemia, diabetes, smoking, sleep apnea, etc.) and treat them aggressively. It is also recommended to investigate lifestyle and physical activity history to define a comprehensive intervention, inclusive of a reduced-calorie diet and increased physical activity. Among the multiple physiopathologic components of obesity inflammation (Vinik et al., 2013), hyperinsulinemia (Emdin et al., 2001), and adipokines (Smith and Minson, 2012) contribute to the development of autonomic dysfunction, variously combining sympathetic over-activity (Grassi et al., 2019) and baroreflex impairment (Skrapari et al., 2007).

The obesity-linked autonomic dysfunction (Costa et al., 2019) may also present various degrees of cardiac autonomic neuropathy (CAN) (Williams et al., 2019), a potentially serious, partly reversible, complication of metabolic conditions that at the extremes of diabetes elevates the risk of death to 3.6 (Vinik and Ziegler, 2007). Early screening for autonomic dysfunction may be clinically advantageous and may be performed using multiple techniques, ranging from the moderately invasive electroneurographic recordings of efferent muscle sympathetic activity (Grassi et al., 1998) to the non-invasive analysis of heart rate variability (HRV) (Katsilambros et al., 2011; Kokkinos et al., 2013) and cardiac baroreflex (Skrapari et al., 2007). This method may also be employed to assess reversibility of autonomic impairment with weight loss (Costa et al., 2019) or physical activity (Voulgari et al., 2013). The complexity of the role of the autonomic nervous system (ANS) in obesity has been recently described (Guarino et al., 2017) and the usefulness of HRV analysis, particularly for early screening, indicated (Williams et al., 2019). The combination of HRV and baroreflex is a powerful tool to assess cardiac risk in various conditions ranging from myocardial infarction (La Rovere et al., 2001) to hypertension (Pagani et al., 1988) or heart failure (Chattipakorn et al., 2007).

Spectral analysis of RR interval variability and cardiac baroreflex (Zilliox and Russell, 2020) could, in particular, furnish a convenient method to assess early phenotypic alterations of cardiac autonomic regulation (CAR), especially the vagal arm (Zygmunt and Stanczyk, 2010), supported by a medium-strong correlation between cardiac baroreflex and several RR variability-derived autonomic indices (Solaro et al., 2019). Proponents of this computational approach (e.g., Pagani, 2000; Bernardi and Bianchi, 2016) pointed out that this method was simpler, less demanding of patients, better streamlined, and ideal for clinical protocols requesting repeated assessments also in real-life conditions, or at a distance.

Data collection and interpretation of analysis represent, therefore, the major steps of autonomic assessment, with a goal of supporting clinical decisions based on these novel patient-oriented biomarkers (Kerkhof et al., 2019). However, it must be noted that HRV provides, as in other clinical fields, like, e.g., cardiology or radiology, measured and calculated quantities, which are considered unequivocally interpretable against reference values (hence ongoing debates). Notably, measured variables are expressed in physical units (ms, kg, m, etc.) while computed (or transformed) variables, often obtained as ratios (e.g., normalized units or LF/HF ratio), assume “pure” numbers having no measurement unit. Focusing on single numbers corresponds to a loss of information. However, the exploitation of their additive value allows combining congruent measures: hidden physiological meaning might become manifest. Transposing this problem into a context where a multitude of HRV and cardiac baroreflex indices, each with its own unit measurement or not, is available, necessarily requires to extract a limited set of statistical indicators (Lucini et al., 2018) derived, e.g., from multivariate statistical analysis (Jobson, 1991). Statistical indicators, reducing data dimensionality, allow more straightforward interpretations of complex phenomena than multiple separate indices. They must nonetheless provide indications that could be of immediate clinical or prevention value (Goossens, 2017) and allow analyses to be carried out as best as possible *ceteris paribus*, e.g., for comparative purposes.

Consequently, the challenge was to set up statistical indicators capable, at the same time, of reducing the complexity of a set of multiple indices, being immediately interpretable in the study field, and being free of individual characteristics that, if not controlled for, could bias analysis conclusions or severely affect comparisons among groups. A possible solution can be pursued by using a mix of statistical methods in an integrated manner, in particular statistical transformations and multivariate statistical analysis techniques (Jobson, 1991). In this specific context, we developed a methodology to cope with the problem of detecting and synthesizing specific traits of CAR. The starting set of variables was given by the multitude of indices obtained by the autoregressive spectral analysis of short-term RR interval and systolic arterial pressure variability (Pagani et al., 1986) that we treated overall as ANS proxies (Lucini et al., 2018; Solaro et al., 2019). These proxies were collected on a population of 756 otherwise healthy subjects together with personal data and BMI.

Subsequently, we transformed the ANS proxies to free them of age and gender bias and obtained so-called *adjusted ANS proxies* (Solaro et al., 2017, 2019). Next, to reduce data dimensionality, we applied the exploratory factor analysis (EFA) (Thompson, 2004), which is one of the most popular multivariate statistical analysis methods (Jobson, 1991). As the main advantage, EFA allows the construction of a small number of uncorrelated common latent factors from the original set of the interrelated variables that may reproduce a good percentage of the observed data variability. In this sense, latent factors can be regarded as statistical indicators. Nonetheless, the main disadvantage is that latent factor values (i.e., factor scores) might not admit straight practical interpretations, even if latent factors link with a precise meaning to the original variables. To overcome this drawback, we applied the percentile rank transformation (Conover and Iman, 1981; Murphy and Davidshofer, 2004) to latent factors in order to obtain synthetic indicators with values ranging from 0 to 100, where, depending on the meaning assigned to each factor, values at the extremes of the range can identify either the better or the worst condition.

Once we composed the set of different CAR indicators, we inspected our study hypothesis that elevated BMI conditions associate significantly with CAR alterations (Molfino et al., 2009) net of age and gender effects. At the same time, we employed the autonomic nervous system indicator (ANSI) introduced by Sala et al. (2017) as a composite indicator of CAR, which is free of age and gender effects by construction. ANSI aims to substitute a multitude of indices in describing the function of a multidimensional control network (Malliani et al., 1991; Fukuda et al., 2015), with the concerted goal of supporting decision-makers, such as physicians and trainers. In a similar way to the other EFA-based indicators, ANSI ranges from 0 to 100 (the higher, the better), so that it immediately furnishes an indicator of the efficiency of CAR (Sala et al., 2017). Then, to inspect our study hypothesis validity, we relied on several different non-parametric inferential testing procedures (Bowman and Azzalini, 1997; Hollander et al., 2014) in order to draw the most reliable inference possible. In particular, we assessed whether there exist significant differences concerning the CAR indicators in the comparisons among the considered BMI groups (i.e., normoweight, overweight, and obese). Parenthetically, we also tested the hypothesis that ANSI might prove useful to assess the impairment of CAR occurring with initial, simple obesity (Guarino et al., 2017) as defined by elevated BMI, independently of age and gender. Besides, by testing the exchange of information (Haken, 1983) between ANSI and the cardiac baroreflex, we provided an additional criterion to evaluate the potential value of this novel monovariate proxy of CAR.

## Materials and Methods

This retrospective, proof of concept study is based on data from the short-term HRV anonymized database of the Exercise Medicine Clinic of the University of Milan that is part of an ongoing project on the feasibility of HRV as an autonomic metric in the management of cardiovascular prevention in outpatients (approved by the Ethical Committee of the University of Milano) (Lucini and Pagani, 2012). We employed data from healthy individuals (*n* = 756 in all, see Table 1) with 283 males and 473 females, and mean age 35.9 ± 12.41 years, with BMI ranging from normal to elevated (overweight and obese). Health status was defined based on their family physician judgment or by clinical history and physical examination. Exclusion criteria comprised: age < 17 years; acute illness within the past 3 months; chronic conditions, particularly those known to alter autonomic regulation (such as diabetes or hypertension). Considering recent guidelines (Jensen et al., 2014), we divided the total population into three different groups according to BMI (Table 1): normoweight group (NW) (BMI < 25 Kg/m^2^), overweight group (OW) (25 ≤ BMI < 30 Kg/m^2^), and obesity group (OB) (BMI ≥ 30 Kg/m^2^). The study conforms to the standards set by the declaration of Helsinki on investigations regarding human subjects. All participants at the time of the Surgery visit had signed an agreement to use anonymized data for population studies.

**TABLE 1:** Frequency and percentage distributions of the BMI groups along with descriptive statistics of BMI, age, and gender within the BMI groups.

**BMI group**	**Definition**	**Count**	**Percentage**	**BMI mean ± SD**	**Age (years) mean ± SD**	**n Male / n Female**
Normoweight (NW)	Subjects with BMI < 25 Kg/m^2^	472	62.4%	21.33 ± 2.10	32.85 ± 11.68	163 / 309
Overweight (OW)	Subjects with 25 ≤ BMI < 30 Kg/m^2^	163	21.6%	26.91 ± 1.36	40.03 ± 12.17	89 / 74
Obese (OB)	Subjects with BMI ≥ 30 Kg/m^2^	121	16.0%	38.28 ± 7.81	42.26 ± 11.49	31 / 90
Total		756	100.0%	25.24 ± 7.09	35.90 ± 12.41	283 / 473

### Cardiac Autonomic Regulation

Assessment of CAR is based on autoregressive spectral analysis (methodological details in the Supplementary Material) of short-term RR and systolic arterial pressure (SAP) variability [as recently summarized in Solaro et al. (2019)]. Briefly, a single-lead ECG (CM5), respiratory activity (piezoelectric belt), and SAP (plethysmographic approach – Finometer Midi, FMS) are continuously recorded over a 5–7 min period at rest and subsequently for additional 5 min, while standing up unaided. The active stand condition is thought to induce a physiological shift of the ANS sympathetic-parasympathetic balance toward a sympathetic predominance mostly due to baroreceptor unloading and depotentiation of the negative feedback baroreflex (Pagani et al., 1986). A series of cardiac autonomic proxies are derived using HeartScope (AMPS, NY, United States), an RR variability autoregressive spectral analysis tool (Table 2; Badilini et al., 2005).

**TABLE 2:** Definition of the variables (ANS proxies) utilized for the study^(a)^.

**Vars.**	**Units**	**Definition**
HR	beat/min	Heart Rate, sinus rhythm from single trace ECG
RR Mean	ms	Average of RR interval from tachogram, discretization to 0.1 ms, sampling frequency 300 Hz or more. If slower, use parabolic interpolation to detect peak R wave
RR TP	ms^2^	RR variance from tachogram
RR LFa	ms^2^	Absolute power(a) of Low Frequency (LF) spectral component of RR variability (V) (frequency range 0.03–0.14 Hz)
RR HFa	ms^2^	Absolute power(a) of High Frequency (HF) spectral component of RRV (frequency range 0.14–0.45)
RR LFnu	nu	Normalized power (nu) of Low Frequency (LF) component of RRV
RR HFnu	nu	Normalized power (nu) of High Frequency (HF) component of RRV
RR LF/HF	—	Ratio between absolute values of LF and HF
RR LFHz	Hz	Center frequency of LF oscillatory component, considering extremes of 0.03–0.14 Hz
RR HFHz	Hz	Center frequency of HF component, normally synchronous and highly coherent (>0.5) with the respiratory series (respirogram), providing a measure of respiratory rate
ΔRR LFnu	nu	Difference in LF power in nu between stand and rest
α index	ms/mmHg	Frequency domain measure of baroreflex gain
SAP	mmHg	Systolic arterial pressure by sphygmomanometer
DAP	mmHg	Diastolic arterial pressure by sphygmomanometer
SAP Mean	mmHg	Average of systogram (i.e. systolic arterial pressure variability by Finometer)
SAP LFa	mmHg^2^	Absolute power of LF component of systogram

*^(a)^Modified from Pagani et al. (1986, 1988), Lucini et al. (2018), Solaro et al. (2019). Notice that HRV (heart rate variability) is commonly and interchangeably used with RRV (RR interval variability). This latter term would be more appropriate when dealing indeed with RR interval metrics. However, the more common term HRV is also used in the present study to indicate the general topic of variability of inter-beats intervals, for simplicity.*

### Statistics

The methodology we developed for setting up synthetic indicators was employed to detect and synthesize specific CAR traits controlling for age and gender effects. These age-and-gender-adjusted statistical indicators were then used as primary references to study our hypothesis that the transition from normoweight to obesity according to BMI associates significantly with alterations in the overall ANS state, as reflected by the ANS proxies. Specifically, the reference point in all the analyses was the *n* = 756 study participants’ subdivision in the three BMI groups defined in Table 1. In particular, the majority of subjects (62.4%) fell into the NW group (with mean BMI 21.33 and mean age 32.85 years), while 21.6% into the OW group (mean BMI 26.91 and mean age 40.03 years), and 16% into the OB group (mean BMI 38.28 and mean age 42.26 years). Regarding the ANS individual state description, we relied on the 16 ANS proxies (HRV and blood pressure measures) listed in Table 2, with a particular focus put on the α index (Lucini et al., 2002).

**FIGURE 1:**
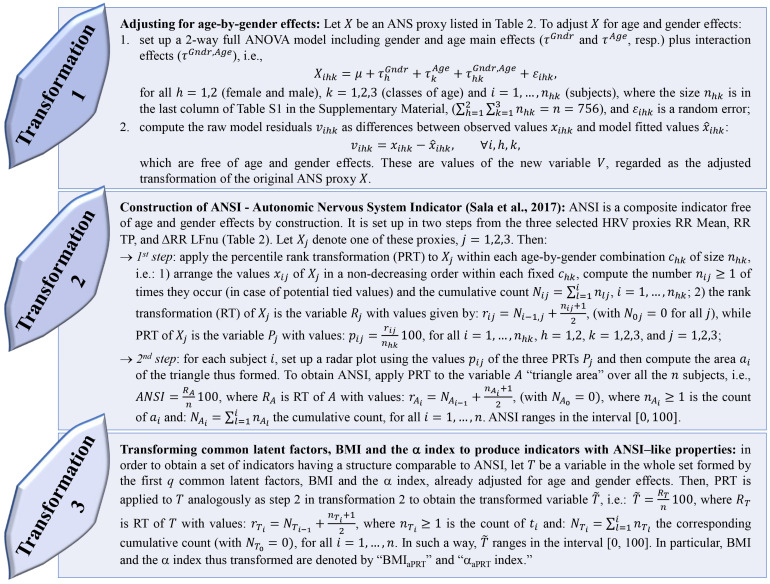
Transformations applied to the original variables to set up CAR indicators.

Regarding the need to set up and work with adjusted statistical indicators, we had to cope with two main issues while meeting the study objective. First, similarly to what already argued in previous works (Solaro et al., 2017, 2019; Lucini et al., 2018), statistical analyses concerning the relationship between the ANS state and the BMI condition required to be carried out under the same combinations of gender and classes of age, i.e., as far as possible *ceteris paribus*. However, stratifying subjects into gender-by-age combinations was not appropriate here because it would have implied forming subgroups too small in size (see Supplementary Table 1 in the Supplementary Material reporting the within-BMI-groups distributions of the 756 subjects in gender-by-age combinations. E.g., there are only three obese males in the age class 17–30 years). A second issue was the need to take into account the multicollinearity among the ANS proxies and then reduce their number according to some optimal criteria and with a limited loss of information. To overcome these drawbacks, we applied several statistical transformations (methodological details in Figure 1) addressed to:

(1) adjusting the ANS proxies for gender and age effects to obtain so-called *adjusted ANS proxies*. The procedure we used, already applied in Lucini et al. (2018) and Solaro et al. (2019), is the transformation 1 described in Figure 1.
(2) setting up a limited number of indicators from the original set of the ANS proxies such that they were free of gender and age effects by construction. To this aim, we relied on two alternative procedures: (a) we applied EFA (with the maximum likelihood extraction method and varimax rotation (Thompson, 2004)) to the set of the 16 adjusted ANS proxies to produce a smaller number of uncorrelated variables that were by nature net of gender and age effects. We kept in analysis the first *q* < 16 common latent factors such that they reproduced each at least 10% of the total variance and at least 15% of the total communality. Then, we interpreted the meaning of the extracted common factors using the factor loadings, i.e., the correlation coefficients between the factors and the adjusted ANS proxies, greater than, or equal to, 0.5 in absolute value; (b) we constructed ANSI (Sala et al., 2017) using the procedure described in the transformation 2, Figure 1. ANSI is a composite indicator based on the three selected HRV measures RR Mean, RR TP, and ΔRR LFnu, and is free of gender and age effects by construction. One of the main advantages is that ANSI, being set up by the percentile rank transformation, ranges over the interval [0, 100], so that the information it carries with it is immediately valuable from a clinical point of view. In particular, higher percentiles correspond to better ANS conditions, while lower percentiles indicate impaired ANS states;
(3) re-expressing a selected subset of variables of interest, i.e., the first *q* extracted common factors, BMI, and the α index, in percentile ranks so that throughout the statistical analyses, their performance could be compared directly to ANSI (transformation 3, Figure 1). Regarding the common factors, despite the meaning assigned to them by factor loadings, their values (i.e., factor scores) might be of no immediate practical interpretation (e.g., common factors, having zero-mean by construction, assume both negative and positive scores). The percentile rank transformation helped overcome this drawback, translating factor scores into percentiles and being interpreted more quickly in clinical terms. We regarded the common latent factors thus transformed as *real* ANS indicators. On the other hand, we assigned ANSI-like properties to BMI and the α index (i.e., gender-and-age-effects adjustment and range in the interval [0, 100]) by applying the percentile rank transformation to the corresponding adjusted variables obtained by transformation 1 in Figure 1. We denoted BMI and the α index thus transformed as “BMI_*aP*__*RT*_” and “α_*aP*__*RT*_ index” (where subscript “aPRT” stands for “adjusted Percentile Rank Transformation), respectively.

The set consisting of the *q* ANS indicators, ANSI, and the α_*a*__*PRT*_ index thus obtained represented the actual set of variables to which we addressed statistical analyses for inspecting their relationship with the BMI categorization into the three groups NW, OW, and OB. For completeness, we also involved BMI in several analyses as a continuous variable. That was why we transformed BMI to assign it ANSI-like properties and obtained the “BMI_*a*__*PRT*_” version of BMI.

**FIGURE 2:**
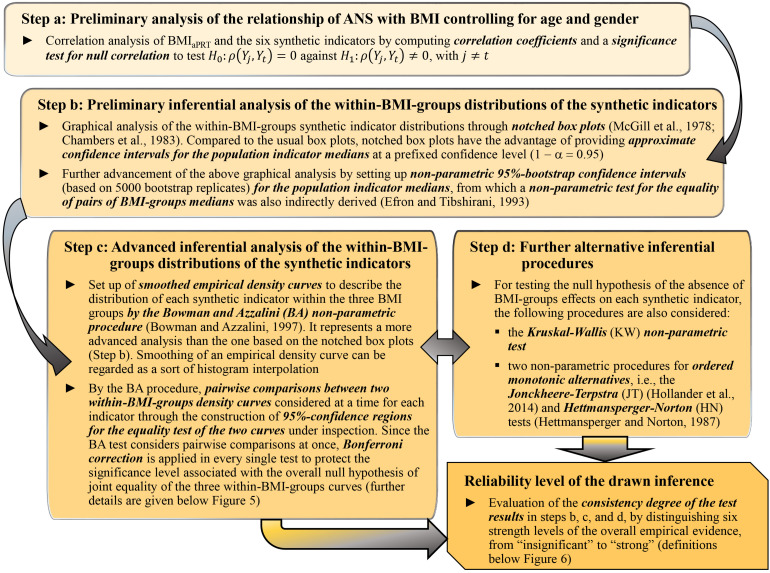
Steps of data-driven statistical analyses performed to study the effects of the BMI categorization in NW, OW, and OB groups on the synthetic indicators.

Statistical analyses were carried out substantially according to a data-driven instead of a model-based approach. At a preliminary, exploratory stage, we studied the mean dependency of each original ANS proxy on the BMI groups through testing procedures, i.e., the usual univariate ANOVA (Jobson, 1991) and the Kruskal–Wallis (KW) non-parametric test (Hollander et al., 2014). We also examined the correlation coefficients of BMI and the ANS proxies and provided the standard significance test for null correlation. After that, we focused extensively on the relationship of the *q* ANS indicators, the α_*a*__*PRT*_ index, and ANSI [denoted in brief as the set of the (*q* + 2) “*synthetic indicators*”] with BMI. The main goal was to infer, net of age and gender, whether the transition from a normoweight to an obesity condition combined with significant changes, as captured by the synthetic indicators, in the ANS state. To this aim, we applied the four analysis steps described in Figure 2 and addressed to study the effects of the BMI categorization in NW, OW, and OB groups on the distribution of the synthetic indicators representing various CAR traits. The main objective of the four analysis steps, applied in the order reported in Figure 2, was to study, by increasing the complexity of the approaches and without *a priori* conjectures on the data distribution, the relationship between BMI and the synthetic indicators through different non-parametric statistical methods. In particular, by the Jonckheere–Terpstra (JT) (Hollander et al., 2014) and Hettmansperger–Norton (HN) tests (Hettmansperger and Norton, 1987) for ordered alternatives (Step d, Figure 2), we intended to assess whether the transition from normoweight to obesity combines with a worsening in the overall ANS condition as captured by the set of synthetic indicators. Then, as the final stage of the analyses, we evaluated the non-parametric test result consistency degree to draw the most reliable possible inference toward our study conjecture. We regarded the observed changes in the ANS states, as captured by the synthetic indicators, as *fairly strongly* linked to the BMI condition transition if the KW, median, BA, JT, and HN test results (Figure 2) were highly consistent. Specifically, we distinguished six strength levels of the overall empirical evidence (from “insignificant” to “strong”; all the definitions are in the legend below Figure 6) according to the consistency degree of the test results and regarded our objective as met in the presence of at least a “medium” level of consistency of the test results.

**FIGURE 3:**
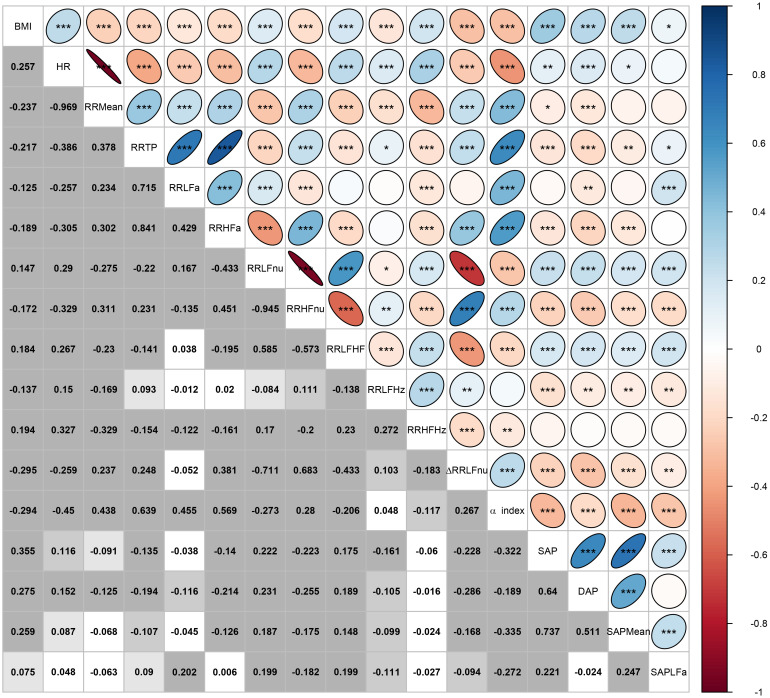
Correlation plot of BMI and the ANS proxies computed over the whole set of subjects. In the lower triangular part of the matrix, displayed values are the Pearson correlation coefficients *r* computed for every pair of variables (*X*_*j*_,*X*_*t*_), *j*,*t* = 1,…,17, and *j*≠*t*. In each cell, background gray shades denote the empirical significance level of each test for null correlation, i.e., *H*_0_:ρ(*X*_*j*_,*X*_*t*_)=0 vs. *H*_1_:ρ(*X*_*j*_,*X*_*t*_)≠0: 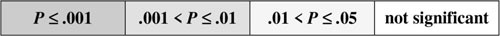 In the upper triangular part, these same correlation coefficients are represented as ellipses including the empirical significance level code internally: ^∗∗∗^ = *P* ≤ 0.001, ^∗∗^ = 0.001 < *P* ≤ 0.01, ^∗^ = 0.01 < *P* ≤ 0.05.

**FIGURE 4:**
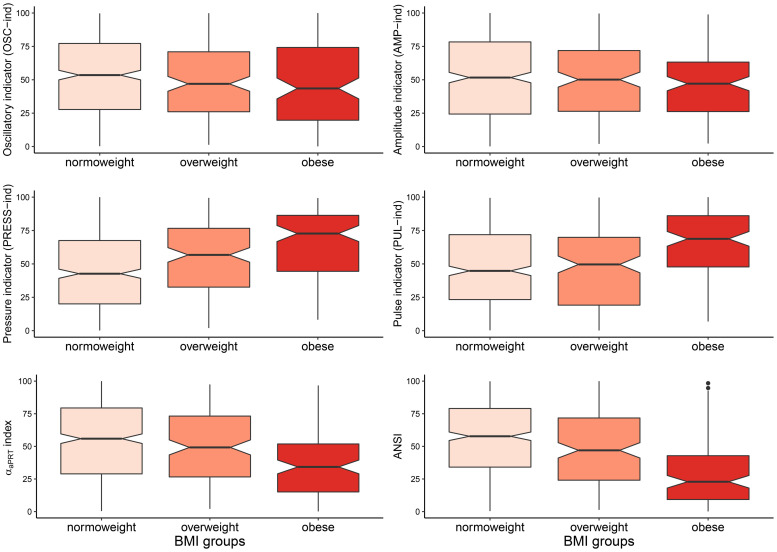
Notched box plots of the distributions of the four ANS indicators, the α_*aPRT*_ index, and ANSI within the BMI groups. Notches around the sample medians *med*_*g*_(*Y*) of the synthetic indicator *Y* within the BMI groups: *g* = normoweight (NW), overweight (OW), obese (OB), are given by the formula: $m\,e\,d_{g}\,\left(Y\right)\pm 1.58\,I\,Q\,R_{g}\,\left(Y\right)/\sqrt{n_{g}}$, where *IQR*_*g*_(*Y*)=*Q*_3,*g*_(*Y*)−*Q*_1,*g*_(*Y*) is the interquartile difference for *Y* within group *g* with sample size *n_g*, and *Q*_*1,g*_ and *Q*_*3,g*_ are the first and third quartiles, respectively (McGill et al., 1978; Chambers et al., 1983; Wickham, 2016). Notches providing an approximate 95%-confidence interval for the corresponding population medians can be used as a convenient graphical tool for comparing pairs of groups. If the notches of the medians of two groups do not overlap, then there may be systematic differences in the population. Notches depicted in the above box plots are not, however, adjusted for simultaneous comparisons of all the three groups together (McGill et al., 1978; Chambers et al., 1983). Only pairwise comparisons are allowed.

**FIGURE 5:**
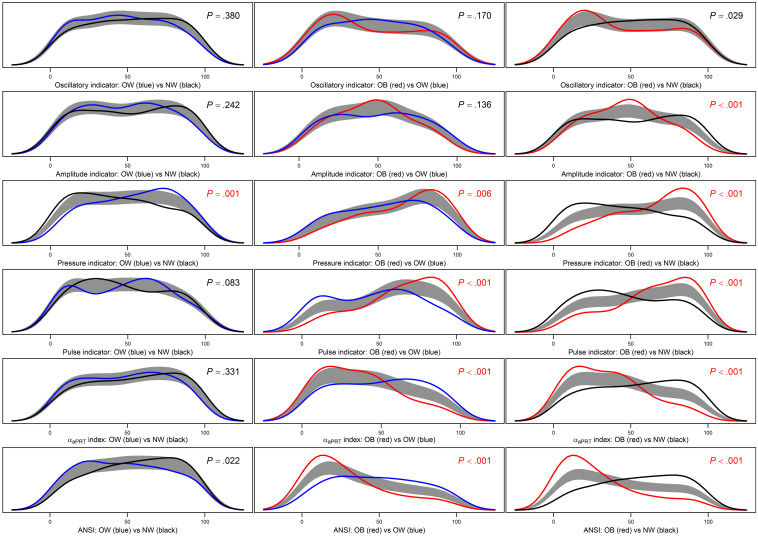
Panel plot of estimated density curves and non-parametric 95%-confidence regions for the pairwise equality of the distributions of the four ANS indicators, the α_*aPRT*_ index, and ANSI within the BMI groups. Density estimates are obtained through the kernel density method by Bowman and Azzalini (1997) in order to have smoothed empirical density curves. In the above panels, the black curve refers to the normoweight group, the blue curve to the overweight group, and the red curve to the obese group. The gray region is a reference band for the equality test of the curves in each pairwise comparison, i.e., $H_{0}:f_{g}\,\left(y\right)=f_{g^{\prime }}\,\left(y\right)$, for all *y*, against: $H_{1}:f_{g}\,\left(y\right)\neq f_{g^{\prime }}\,\left(y\right)$, for at least one *y*, where *f*_*g*_(*y*) and $f_{g^{\prime }}\,\left(y\right)$ are population density functions of the synthetic indicator *Y* within the BMI groups *g* and *g*′, respectively, with *g*≠*g*′= NW, OW, and OB. *P*-values of the Bowman and Azzalini (BA) test are reported in each panel. A Bonferroni correction is applied to the nominal significance level α=0.05 to preserve the significance level of the overall null hypothesis *H*_0_:*f*_*NW*_(*y*)=*f*_*OW*_(*y*)=*f*_*OB*_(*y*), for all *y*. Because there are three distinct pairwise comparisons for each indicator *Y*, the significance level applied to each BA test is: $\alpha^{*}=\frac{0.05}{3}=0.017$. *P*-values resulted as lower or equal to α^∗^ are written in red.

We performed all the statistical analyses with the R software, version 3.6.1 (R Core Team, 2019), together with the R contributed packages: “corrplot” for the correlation plot in Figure 3 (Wei and Simko, 2017); “DescTools” for the non-parametric bootstrap confidence intervals of the medians and the JT test (Signorell et al., 2019); “ggplot2” for the notched box plots in Figure 4 (Wickham, 2016); “pseudorank” for the HN test (Happ et al., 2018); “psych” for factor analysis (Revelle, 2018); “sm” for the smoothed empirical density curves in Figure 5 and the BA test (Bowman and Azzalini, 2018).

## Results

Descriptive data concerning the 16 ANS proxies listed in Table 2 are presented in Table 3 in the form of total and within-BMI-groups means and standard deviations. Table 3 highlights the presence of quite expected monotonic trends of the within-BMI-groups means as the groups vary from NW to OB. In particular, the OB group is characterized by the highest means of HR, RR LFnu, RR LF/HF, RR HFHz, SAP, DAP, and SAP Mean, and the lowest means of RR Mean, RR TP, RR LFa, RR HFa, RR HFnu, RR LFHz, ΔRR LFnu, and the α index. The NW group has the opposite characteristics, while the OW group represents an intermediate condition (excepted SAP LFa, whose mean is the highest). Overall, the ANOVA and KW tests give empirical evidence of BMI-groups effects on the population ANS proxy means, except for SAP LFa. For instance, by both ANOVA and KW tests, at least two BMI-groups effects on the α index result significantly different (*P* < 0.001). The other test results can be read similarly.

**TABLE 3:** Descriptive data (mean and standard deviation) of the ANS proxies within the BMI groups and over the whole set of subjects.

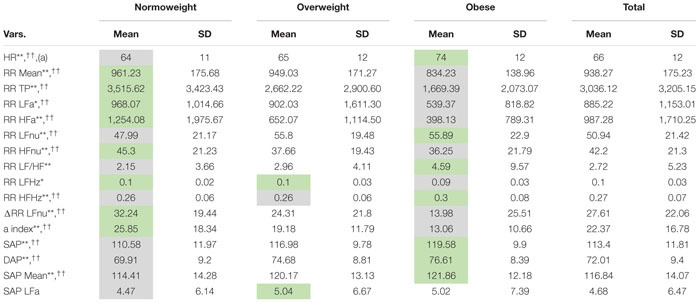

^(a)^HR means and standard deviations are rounded to integers. In every comparison among the within-BMI-groups means of each variable, a green cell denotes the largest computed mean, while a gray cell denotes the smallest computed mean. Univariate parametric ANOVA (based on the F test) and Kruskal–Wallis test are alternative inferential procedure used to verify, for each ANS proxy, the null hypothesis of equality of BMI-groups effects: *H*_*0*_: τ_*NW*_ = τ_*OW*_ = τ_*OB*_ against the alternative hypothesis: *H_1*: τ_*g*_ ≠ $\tau_{g^{\prime }}$ for at least one pair of groups (*g*,*g*′), with *g*≠*g*′= normoweight (NW), overweight (OW), obese (OB), and where τ_*g*_ is the effect of group *g*. Empirical significance level code in the univariate parametric ANOVA: ** = *P* ≤ 0.001, * = 0.01 < *P* ≤ 0.05. Empirical significance level code in the Kruskal–Wallis test: †† = *P* ≤ 0.001.

Figure 3 encloses all the analyses regarding the correlation between BMI and the ANS proxies. Two remarks are worth making. First, by various intensity and sign, BMI correlates significantly with all the ANS proxies (first row and first column in Figure 3). The highest correlation coefficients (in absolute value) concerning BMI are observed, positively, with the blood pressure measures SAP, DAP, and SAP Mean (0.355, 0.275, and 0.259, respectively, with *P* < 0.001), and the Heart Rate measure (0.257, *P* < 0.001), and negatively with ΔRR LFnu (−0.295, *P* < 0.001) and the α index (−0.294, *P* < 0.001). This finding further suggests meaningful connections between the BMI conditions and at least a subset of the ANS proxies. Second, as expected, the ANS proxies also are inter-correlated by a very different intensity and sign. For instance, RR LFnu, RR HFnu, and ΔRR LFnu correlate pairwise to a great extent. The correlation coefficient of RR LFnu and RR HFnu is strongly negative (−0.945, *P* < 0.001), as well as the correlation coefficient of RR LFnu and ΔRR LFnu (−0.711, *P* < 0.001). However, the correlation coefficient of RR HFnu and ΔRR LFnu is strongly positive (0.683, *P* < 0.001). On the other hand, the α index and RR LFHz are almost uncorrelated (0.048, *P* = 0.187), as are ΔRR LFnu and RR LFa (−0.052, *P* = 0.155). That further supports the need for reducing the numeric complexity of the whole set of the intertwined ANS proxies.

Although valuable from an exploratory point of view, the above analyses concerning the mean dependency of the ANS proxies on the BMI groups and the correlation of BMI and the ANS proxies suffer from a main critical point, i.e., they do not control for the effects of the fundamental biological parameters represented by age and gender. Accordingly, we have applied transformation 1 described in Figure 1 to obtain the ANS proxies adjusted for age and gender effects. By box plots of the within-BMI-groups distributions of both the original and the adjusted ANS proxies (see Supplementary Figure 1 in the Supplementary Material), specific trends in the original ANS proxy distributions can be observed with the varying from NW to OB, and the adjusted ANS proxies share these trends. That is a crucial point in that it suggests the presence of meaningful connections of the various ANS states with the BMI conditions even when age and gender effects are taken under control in the analyses.

The next step is constructing a limited number of indicators from the original set of the ANS proxies to reduce the ANS complexity and consider their reciprocal interrelations. We have then applied EFA to the 16 adjusted ANS proxies to produce latent factors that are naturally adjusted for age and gender effects. Table 4 displays the factor loadings of the first *q* = 4 common latent factors, which are kept in the analysis because they jointly satisfy the two criteria fixed *a priori*, i.e., reproducing each at least 10% of the total variance and at least 15% of the total communality. By Table 4, the first four common factors, which reproduce together 60.62% of the total variance, reveal clusters of multiple adjusted ANS proxies that link to a smaller number of hidden components carrying a similar meaning.

**TABLE 4:** Factor analysis with the maximum likelihood extraction method arrested to the first four factors: rotated factor loadings with the varimax method.

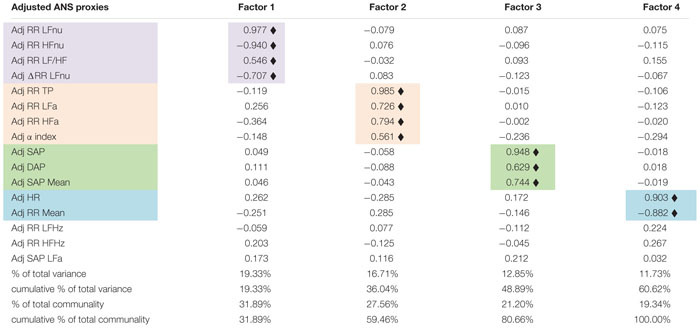

*Total communality (i.e., total reproduced variance) = 9.699, total variance = 16, percentage of total variance explained = 60.62%. Printed values are correlation coefficients between the adjusted ANS proxies and the first *q* = 4 common latent factors. Black lozenges flag correlation coefficients greater than, or equal to, 0.5 in absolute value. Interpretation underlying the first four factors: Factor 1 = oscillatory domain (variables colored in lilac), Factor 2 = amplitude domain (variables in pink), Factor 3 = pressure domain (variables in green), Factor 4 = pulse domain (variables in blue).*

In detail, factor 1 (nearly 19% of total variance and 32% of total communality) relates to the normalized oscillatory information. Because it correlates (highly) positively with adjusted RR LFnu and RR LF/HF, and negatively with adjusted RR HFnu and ΔRR LFnu, it represents the oscillatory domain. Factor 2 (about 17% of total variance and 28% of total communality) links to the absolute amplitude domain for its high positive correlations with adjusted RR TP, RR LFa, RR HFa, and the α index. Factor 3 (about 13% of total variance and 21% of total communality) relates to the pressure domain because of the high positive correlations with adjusted SAP, DAP, and SAP Mean. Finally, factor 4 (nearly 12% of total variance and 19% of total communality) expresses the pulse variations given the high positive correlation with adjusted HR and the high negative correlation with adjusted RR Mean. It represents, therefore, the pulse domain.

After that, we have set up the six following synthetic indicators for the arguments previously advanced. These indicators represent the central reference in the subsequent analyses addressed to prove our study conjecture. In detail, we have re-expressed the extracted four common latent factors in terms of real ANS indicators by applying transformation 3 in Figure 1. In such a way, we have obtained the oscillatory indicator (OSC-ind) from factor 1, the amplitude indicator (AMP-ind) from factor 2, the pressure indicator (PRESS-ind) from factor 3, and the pulse indicator (PUL-ind) from factor 4. We have also applied the same transformation 3 to the α index and obtained the α_*a*__*PRT*_ index. At the same time, we have set up ANSI (transformation 2 in Figure 1) by using three selected ANS proxies, each linking to a different factor (Table 4), i.e., ΔRR LFnu to factor 1 (oscillatory domain), RR TP to factor 2 (amplitude domain), and RR Mean to factor 4 (pulse domain).

The last stage of the statistical analyses regards the in-depth examination of the six synthetic indicators within the BMI groups according to the four analysis steps in Figure 2. Specifically:

*Step a:* Table 5 reports the *correlation coefficients* of the six synthetic indicators in addition to BMI_*aPRT*_, i.e., BMI re-expressed according to transformation 3 in Figure 1. Several remarks are worth making. First, all the correlation coefficients result as significant. Second, BMI_*aPRT*_ correlates moderately and positively with PRESS-ind (0.293, *P* < 0.001), and moderately and negatively with the α_*a*__*PRT*_ index (−0.252, *P* < 0.001) and ANSI (−0.279, *P* < 0.001), [note in particular that the correlation coefficient between the original BMI and α index is −0.294, *P* < 0.001, (Figure 3)]. That evidences that, net of age and gender, higher BMI values tend to connect to higher values of blood pressure measures, on the one hand, and to lower values of the baroreflex gain index and ANSI, on the other hand. In other words, this approach also shows that obesity tends to associate significantly with altered ANS states. Third, the correlation coefficient of the α_*a*__*PRT*_ index and ANSI is high and positive (0.646, *P* < 0.001). Fourth, in their turn, the α_*a*__*PRT*_ index and ANSI correlate at least moderately with most of the four ANS indicators. In particular, as expected, the correlation of the α_*a*__*PRT*_ index and AMP-ind is positive and medium-high (0.580, *P* < 0.001) because the adjusted α index is one of the variables that have contributed to the set-up and the meaning of factor 2 (Table 4). Similarly, ANSI correlates medium-highly with OSC-ind (0.459, *P* < 0.001), AMP-ind (0.555, *P* < 0.001), and PUL-ind (−0.565, *P* < 0.001), since, as already mentioned, it originates from the three selected ANS proxies, each linking to the common latent factor underlying its corresponding ANS indicator.

**TABLE 5:** Correlation coefficients of BMI_*aPRT*_, the α_*aPRT*_ index, and ANSI with the four ANS indicators, and *p*-values of the tests for null correlation.

**Indicators**	**OSC-ind**	**AMP-ind**	**PRESS-ind**	**PUL-ind**	**α_*aPRT*_ index**	**ANSI**
BMI_*aPRT*_	−0.071 *P* = 0.050*	−0.154 *P* < 0.001***	0.293 *P* < 0.001***	0.149 *P* < 0.001***	−0.252 *P* < 0.001***	−0.279 *P* < 0.001***

	**OSC-ind**	**AMP-ind**	**PRESS-ind**	**PUL-ind**	**BMI_*aPRT*_**	**ANSI**

α_*aPRT*_ index	0.178 *P* < 0.001***	0.580 *P* < 0.001***	−0.267 *P* < 0.001***	−0.357 *P* < 0.001***	−0.252 *P* < 0.001***	0.646 *P* < 0.001***

	**OSC-ind**	**AMP-ind**	**PRESS-ind**	**PUL-ind**	**BMI_*aPRT*_**	**α_*aPRT*_ index**

ANSI	0.459 *P* < 0.001***	0.555 *P* < 0.001***	−0.150 *P* < 0.001***	−0.565 *P* < 0.001***	−0.279 *P* < 0.001***	0.646 *P* < 0.001***

Meaning of the labels of the four ANS indicators: OSC-ind = oscillatory indicator (from factor 1); AMP-ind = amplitude indicator (from factor 2); PRESS-ind = pressure indicator (from factor 3); PUL-ind = pulse indicator (from factor 4). Because the synthetic indicators are expressed as percentile scores, Pearson correlation coefficients coincide with Spearman correlation coefficients for rank variables. Correlation coefficients between the indicators generated by factor analysis, i.e., OSC-ind, AMP-ind, PRESS-ind, and PUL-ind, are not displayed because these indicators are uncorrelated by construction (in the same way as the original factors, Table 4). Significance codes of the test for the null hypothesis *H*_0_:ρ(*Y*_*j*_,*Y*_*t*_)=0 against *H*_1_:ρ(*Y*_*j*_,*Y*_*t*_)≠0, with *j*≠*t*: *** = *P* ≤ 0.001, ** = 0.001 < *P* ≤ 0.01, * = 0.01 < *P* ≤ 0.05. Test statistic is given by the Spearman statistic with a Student *t* asymptotic distribution.

*Step b:* Figure 4 displays the *notched box plots* of the within-BMI-groups distributions of the six indicators. With the exceptions of OSC-ind and AMP-ind (first row of panels, Figure 4), the other four indicators exhibit clear trends when moving from NW to OB, i.e., boxes are depicted at, respectively, increasing percentiles for PRESS-ind and PUL-ind, and decreasing percentiles for the α_*a*__*PRT*_ index and ANSI. In particular, in the PRESS-ind case (first panel, second row), notches around the BMI-groups medians (which provide approximate 95%-confidence intervals for the population medians; see legend below Figure 4) do not overlap in any comparison between pairs of groups. A similar trend is seen for ANSI (second panel, third row), although it is less apparent in comparing NW and OW. On the other hand, regarding the α_*a*__*PRT*_ index and PUL-ind, notches around the medians overlap in comparing NW and OW, thus indicating no significant differences between these two group medians. Nonetheless, in both cases, notches do not overlap in the comparisons NW vs. OB and OW vs. OB.

Moreover, Table 6 reports the *non-parametric 95% bootstrap confidence intervals* for the within-BMI-groups indicator medians. That is a more advanced analysis compared to the notches around the medians depicted in Figure 4. Results of the *non-parametric bootstrap median test* are also displayed (in the “Mt” columns). This further analysis fully confirms the above remarks apropos of PRESS-ind and ANSI. Neither of the confidence intervals overlaps, thus proving significant differences in median between every two BMI groups compared at a time. For example, the non-overlapping confidence intervals for the ANSI medians, which are equal to: [53.97, 61.84] in group NW, [39.82, 53.04] in group OW, and [17.33, 28.37] in group OB, evidence how obesity associates with a significant worsening in the ANS state, as captured by ANSI. Regarding the other indicators, the PUL-ind medians are significantly different in the comparisons NW vs. OB and OW vs. OB, but not in NW vs. OW. That is in line with what has been already observed in the pertaining notched box plots (Figure 4). Moreover, the only significant comparison of the α_*a*__*PRT*_ index turns out to be NW vs. OB, while OSC-ind and AMP-ind do not produce any significant pairwise difference.

**TABLE 6:** Non-parametric 95%-bootstrap confidence intervals (CIs) for the medians (5,000 bootstrap replicates) of the four ANS indicators, the α_*aPRT*_ index, and ANSI within the BMI groups, and non-parametric bootstrap median test (Mt) for pairwise equality of the BMI-groups medians.

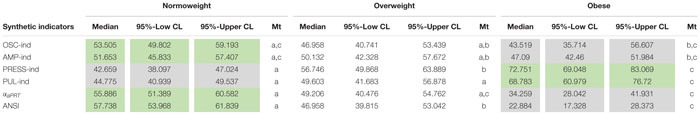

*In every comparison among the within-BMI-groups medians of each synthetic indicator, a green cell denotes the largest computed median (along with its CI), while a gray cell denotes the smallest computed median (plus its CI), (CL is confidence limit). Non-parametric bootstrap median test (columns “Mt”): Let θ_*g*_ be the effect of BMI group *g* on the population median *Med*_*g*_(*Y*) of the synthetic indicator *Y*, with *g* = normoweight (NW), overweight (OW), obese (OB). The null and alternative hypotheses tested by the Mt procedure at the 0.05 significance level are: $H_{0}:M\,e\,d_{g}\,\left(Y\right)=M\,e\,d_{g^{\prime }}\,\left(Y\right)$ against $H_{1}:M\,e\,d_{g}\,\left(Y\right)\neq M\,e\,d_{g^{\prime }}\,\left(Y\right)$ for each pair *g*≠*g*′ = NW, OW, and OB. This significance test is drawn indirectly from the above CIs compared pairwise. In particular, if two CIs overlap then *H_0* is accepted, otherwise *H_0* is rejected at the 0.05 level. Letters “a,” “b,” and “c” in the three columns “Mt” denote the test results obtained in the three distinct pairwise comparisons. Where the same letter appears in two different “Mt” columns, then the corresponding two CIs overlap and *H_0* is accepted. Vice versa, where a letter appears in only one “Mt” column, then the corresponding CI overlaps with neither of the other CIs and *H_0* is rejected at the 0.05 level in favor of *H_1*.*

*Step c:* Figure 5 displays the results of the *BA non-parametric procedure.* It is applied to obtain *smoothed density curves* (an advancement to the box plots) of the within-BMI-groups synthetic indicator distributions and compare them considering two BMI groups at a time. Moreover, the *p*-values of the *BA tests* related to these comparisons are reported inside the panels and are written in red if they result lower than, or equal to, the nominal significance level corrected by the Bonferroni method (i.e., α^∗^ = 0.017). It is immediate to notice that PRESS-ind is the only indicator for which the comparisons are all significant. In the other cases, PUL-ind, the α_*a*__*PRT*_ index, and ANSI have significant results in the two comparisons NW vs. OB and OW vs. OB, while AMP-ind only in the comparison NW vs. OB. Finally, no comparison is significant in the OSC-ind case. Besides this, it is interesting to note the reciprocal position of the curves in the various panels. For instance, by looking at the last panel, third row, in Figure 5, the PRESS-ind NW (black) curve is positively skewed, while the OB (red) curve is negatively skewed. In other terms, subjects in the NW group tend to have lower percentiles on PRESS-ind, while subjects in the OB group higher percentiles. On the other hand, the α_*a*__*PRT*_ index and ANSI (fifth and last rows in Figure 5) share a similar trend in the comparison NW vs. OB (last panels), since now the NW (black) curve is negatively skewed, while the OB (red) curve is positively skewed. Therefore, subjects in the NW group tend to have higher percentiles of the α_*a*__*PRT*_ index and ANSI, while subjects in the OB group lower percentiles.

**FIGURE 6:**
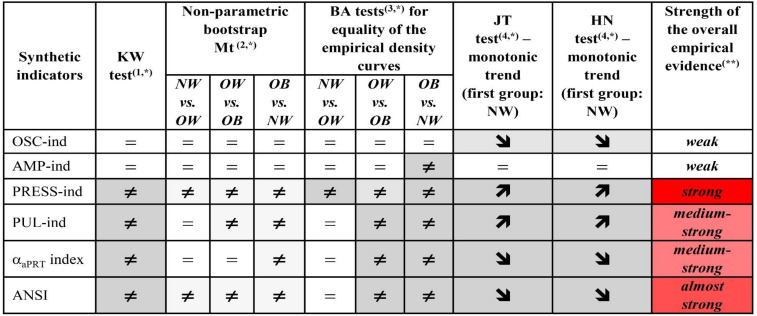
Synoptic figure summing up the results of the Kruskal–Wallis (KW) test, non-parametric bootstrap median test (Mt), Bowman–Azzalini (BA) test, Jonckheere–Terpstra (JT), and Hettmansperger–Norton (HN) tests for ordered monotonic alternatives. Let θ_*g*_ be the effect of BMI group *g* on the synthetic indicator *Y*, with *g* = NW, OW, OB. For each test, the null and alternative hypotheses along with symbols in the above cells, indicating either acceptance or rejection of the null hypothesis, are: ^(1)^ KW test: *H*_*0*_: θ_*NW*_ = θ_*OW*_ = θ_*OB*_ (symbol=) against: *H_1*: θ_*g*_ ≠ $\theta_{g^{\prime }}$ (symbol **≠**) for at least one pair of groups (*g*,*g*′), with *g*≠*g*′; ^(2)^ Median test (Mt): see the legend below Table 6, with *H*_*0*_: $M\,e\,d_{g}\,\left(Y\right)=M\,e\,d_{g^{\prime }}\,\left(Y\right)$ (symbol=) and *H_1*: *Med*_*g*_(*Y*) ≠ $M\,e\,d_{g^{\prime }}\,\left(Y\right)$ (symbol**≠**), with *g*≠*g*′; ^(3)^ BA test: see the legend below Figure 5, with $H_{0}:f_{g}\,\left(y\right)=f_{g^{\prime }}\,\left(y\right)$ (symbol=) and *H*_1_:*f*_*g*_(*y*) ≠ $f_{g^{\prime }}\,\left(y\right)$ (symbol**≠**), with *g*≠*g*′; ^(4)^JT and HN tests: *H*_0_:θ_*NW*_=θ_*OW*_=θ_*OB*_ (symbol=) against the two ordered monotonic alternatives: (a) *H*_1_:θ_*NW*_≤θ_*OW*_≤θ_*OB*_ (symbol ↗), and: (b) *H*_1_:θ_*NW*_≥θ_*OW*_≥θ_*OB*_ (symbol ↘), with at least a strict inequality.^(*)^Background color shades for the significance level of each single test: 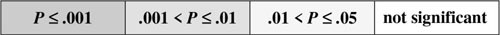 ^(**)^Strength of the overall empirical evidence: *strong* = all the tests are significant; *almost strong* = a test only is not significant; *medium-strong* = KW test is significant and at least one median test plus one BA test are significant followed by both significant JT and HN tests; *medium* = at least one among KW, median, and BA tests is significant followed by either significant JT or HN test (case not occurred here); *weak* = either KW/median/BA tests are significant or JT/HN tests are significant; *insignificant* = no test is significant (case not occurred here).

*Step d:* Results of the *KW test* and the *JT and HN tests for ordered alternatives* are given in Figure 6 (second column and third last and second last columns, resp.). The KW test results are all significant. Regarding the JT and HN tests, we notice that in all the cases, but AMP-ind, there is empirical evidence toward the presence of BMI effects on the synthetic indicators that are either increasing (PRESS-ind and PUL-ind) and decreasing (OSC-ind, the α_*a*__*PRT*_ index, and ANSI). That further confirms most of the remarks already made. Net of age and gender, one can observe an overall progressive worsening in ANS, as captured by the considered indicators, when moving from the NW to OB groups, and ultimately, the existence of an altered ANS state in the presence of obesity.

As the final stage of the analysis, Figure 6 sums up also the results of the performed non-parametric median and BA tests (Table 6 and Figure 5, resp.). The last column in Figure 6 reports, for each indicator, the *level of strength of the overall empirical evidence* in proving the presence of BMI-groups effects on the synthetic indicator distributions. It is worth observing that PRESS-ind has associated the strongest level of consistency of the test results, i.e., all the tests agree in revealing the presence of significant BMI-groups effects controlling for age and gender. ANSI has the second-highest level of strength, the α_*a*__*PRT*_ index and PUL-ind the third one, while OSC-ind and AMP-ind turn out, overall, to have a weak level of strength. Summing up, controlling for age and gender effects, the blood pressure domain, through PRESS-ind, shows the highest tendency for alterations to a worsening in the BMI condition, as well as the overall ANS state results adversely affected by obesity, as assessed on ANSI firstly, and on the α_*a*__*PRT*_ index and PUL-ind secondly.

## Discussion

This retrospective, proof of concept, observational study on a population of otherwise healthy subjects shows that a set of synthetic statistical indicators can be employed to describe the CAR impairment associated with the progressive increase of BMI from normal to obesity, controlling for age and gender. Besides the PRESS-ind, ANSI has proved to be particularly sensitive to BMI increments and tends to capture overweight subjects’ intermediate condition. In addition, the medium-strong correlation between ANSI and cardiac baroreflex further supports the contention that this composite, unitary, monovariate indicator might be used in practice as a proxy of CAR.

### Obesity and Altered CAR

In keeping with the well-known association of obesity with CAR alterations, such as an increase in efferent sympathetic activity, various raw or transformed indices of RRV appear significantly different in the three weight classes, globally indicating autonomic impairment. However, it is difficult to ascribe specific functional meaning to each index or compare various individuals or conditions, also considering the interrelated significant bivariate correlations (Figure 3). Conversely, the progressive decrement of the composite ANSI values from NW to OW and up to OB unquestionably indicates an impairment of CAR, simultaneously providing a standardized measure of the extent of impairment that can be confronted longitudinally considering multiple measurements in the same subject or cross-sectionally in several subjects.

That is a key clinical point, resulting from the novelty that a single (percentile-ranked) indicator of CAR (Sala et al., 2017) may be used to synthesize the autonomic information distributed across the multitude of RRV derived variables (Table 2) commonly employed to (simplistically) separate sympathetic and parasympathetic activity.

It is also important to recall that modern neurophysiological investigations are unraveling the importance of networks (Stam, 2014) that could be extended to visceral nervous activity (Jänig, 2016). Accordingly, it is impossible to distinctly separate various afferent and efferent pathways working together with central structures in “synergistic coordination” (Malliani et al., 1991). In keeping with the original unitary model proposed by Hess (1949), a cybernetic, dual-channel, input-output structure “uses” the dual opposite efferent pathways to govern the heart, in order to correspond, beat-by-beat, to the dynamic requests of the periphery. Such a structural complexity, compounded with the presence of RRV derived parameters that are both reported as physical quantities or else pure “numbers” (Kerkhof et al., 2019), is operationally well served by the unitary index ANSI. In fact, the use of ANSI, in addition to being independent of age and gender, permits to recover the potential loss of information resulting from the difference of autonomic metrics from RRV (Kerkhof et al., 2019), and even improve clinical usefulness, thanks to immediate quantitative (percentile-ranked) evaluation of CAR.

### Statistical Indicators and Clinical Implications

Utilization of HRV derived proxies of CAR in a clinical setting is hampered by several aspects ranging from uncertainties regarding measurement techniques to attribution of individual index meaning. Moreover, autonomic proxies are sensitive to age and gender. All these limitations are overcome by using synthetic statistical indicators built free of age and gender effects (Solaro et al., 2017, 2019; Lucini et al., 2018). In particular, ANSI recapitulates all the information carried by the principal CAR domains (Lucini et al., 2018) through the physical or numerical value attributed to indicators and has, besides, a medium-strong correlation with cardiac baroreflex (Solaro et al., 2019).

The percentile rank transformation applied to all the synthetic statistical indicators (Figure 4) is expected to simplify the attribution of different autonomic regulation levels to individuals and clinical interest groups. Nonetheless, a few statistical technical aspects must be considered. First, as well-known in statistics (see, e.g., Murphy and Davidshofer, 2004, Chap. 5), the percentile rank transformation, re-expressing values of variables into ranks (and subsequently transforming ranks into percentiles), loses the interval (and then the scale) property of variables that are measured at interval (or scale) level. Differences (or ratios) between variable values in the original scale are not preserved because a metric system is replaced by a ranking system formulated in percentile terms. Second, strictly related to the previous remark, the percentile rank transformation tends to amplify small differences of values at the center of the variable distribution and compress large differences in the distribution tails (Murphy and Davidshofer, 2004). Third, by construction, rank-transformed variables are uniformly distributed over the entire support, so they cannot meet, even approximately, the assumption of normality, which is the basic theoretical assumption required in the application of many parametric statistical inferential procedures (e.g., Student *t*-test for equality of the means between two populations, or for nullity of parameters in a linear regression model).

However, while acknowledging these drawbacks, we opted *all the same* for a rank transformation procedure (according to transformations 2 and 3 in Figure 1) in building the six synthetic indicators in Figure 4 (i.e., the four EFA-based ANS indicators, the α_*a*__*PRT*_ index, and ANSI) for the following reasons. In the spirit of the study, we did not intend to set up statistical indicators addressed to *measuring* various CAR facets (in particular autonomic activity). We argue that this should be part of a more ambitious project requiring, before everything else, a larger number of subjects with a broader spectrum of different characteristics. Indeed, while dealing with cross-sectional data, we intended above all to capture the transition process underlying CAR when moving from a control group (i.e., normoweight subjects) to a special-interest group (i.e., obese subjects) through an intermediate-condition group (i.e., overweight subjects). From this perspective, the statistical indicators we introduced should then be regarded more appropriately as *process indicators* (Ahn et al., 2006) rather than measure indicators, while the studied BMI groups-effects as determinants contributing to a transition process. At the same time, the main limitations advanced for the percentile rank transformation can even be regarded as *good* properties because instrumental in unraveling the transition process. Specifically, the loss of the metric property is not a matter of concern here because we were not interested in providing quantitative measures of CAR impairment in overweight and obese subjects compared to normoweight subjects or in quantifying how much different the overall ANS state is (e.g., on average) in the obese compared to the normoweight subjects. The problem of measuring alterations in CAR and the consequent development of CAR statistical measure indicators is a demanding challenge beyond the scope of the study.

Strictly connected to the above remarks, we involved BMI in the analyses as a grouping rather than a continuous variable because we intended to detect the CAR transition process as the BMI states change. Nonetheless, we also performed correlation analyses with the original BMI (Figure 3) and the transformed BMI_*aPRT*_ (Table 5) to have additional insights into the linear relationships between BMI and the other variables. Parenthetically, employing BMI as a grouping variable is the closest approach to clinical practice. It is routine to examine excess weight problems according to BMI categorizations based on standard thresholds.

Moreover, since our focus was put on the transition process underlying the CAR impairment rather than its measurement, we needed a statistical tool capable of reducing the magnitude of large differences of values, which mostly lie in the distribution tails, and simultaneously magnifying small differences of values, which mostly lie in the middle of the distribution. In other words, we intended to put extreme conditions and intermediate conditions on the same level, thus using a sort of magnifier for better capturing the transition at the intermediate state. Otherwise, the highest values overall observed at the opposite BMI groups (i.e., NW and OB) for the individual ANS states would have masked the intermediate BMI group (i.e., OW). Parenthetically, it is worth noting that the percentile rank transformation has the advantage of producing transformed variables (i.e., here the synthetic indicators) that are robust to the presence of outlying subjects. In general, that means that subjects with characteristics extremely far from the main core of data do not severely affect the analyses and do not require being dropped out from data.

Lastly, the statistical approach employed here is essentially data-driven and non-parametric, i.e., distribution-free. We aimed to extract the highest degree of informative content without constraining the data within *a priori* formulated conjectures, in particular distribution assumptions, that could have been too restrictive or, more seriously, could have put us on the wrong track. Consequently, the non-normality of the six statistical indicators is not a matter of concern in this study because all the statistical testing procedures we applied to them, whose results are schematically summed up in Figure 6, are distribution-free. It is worth noting that using the percentile rank transformation as a basis for the construction of statistical process indicators could be ascribed to the general approach by Conover and Iman (1981). The authors regarded data-rank-transforming before carrying out statistical analyses as a possible way to develop new non-parametric statistical procedures.

As further remarks, it is worth stressing that the strength of the overall empirical evidence assigned to the six statistical indicators in the last column of Figure 6 concerns both their reliability and sensitivity in catching CAR alterations across the *whole* transition from the NW to the OB groups passing by the OW group. From a clinical perspective, that means that these process indicators can be regarded as *real* pathophysiological indicators capable of detecting, *as best as possible*, independently of age and gender, the underlying physiological mechanisms that are affected more severely by excess weight up to obesity. Overall, the indicators that have proved to be more responsive to changes in the BMI groups are, first, PRESS-ind, second, ANSI, and finally, at the same level, the α_*a*__*PRT*_ index and PUL-ind. Hence clinically, these findings prove, first of all, that progression to obesity has a strong tendency to contribute to the onset of hypertension as its primary pathophysiological reaction, independently of age and gender. Regarding, in particular, the direct comparison NW vs. OB, the last panel in the third row of Figure 5 provides an effective representation of the impact of obesity on the pressure domain. The PRESS-ind red curve of group OB presents higher ordinates in correspondence to higher percentiles in the abscissa, while the black curve of group NW tends to have an opposite shape (i.e., higher ordinates with lower percentiles), thus showing that obesity tends overall to impair individual pressure conditions. There is also meaningful empirical evidence that the progressive development of obesity adversely affects the overall ANS state (almost strong evidence), along with cardiac baroreflex regulation and pulse variations (medium-strong evidence). These findings have clear pictures regarding NW vs. OB in the last column of panels in Figure 5. For instance, the two ANSI curves depicted in the panel in the last row and column have higher ordinates, respectively, in correspondence to lower percentiles for group OB (red curve) and higher percentiles for NW group (black curve), thus evidencing that obesity tends overall to impair individual ANS states. Similar remarks hold for cardiac baroreflex regulation and pulse variations.

### Limitations

Limitations must be considered. This is an observational study using indirect CAR proxies and involving otherwise healthy subjects, in which the observed male obese group is of small size. However, it should be recalled that spectral oscillations of direct measures of Muscle Sympathetic Nerve Activity (MSNA) in humans show a remarkable correlation with like oscillations in RR V and arterial pressure. That suggests that “normalized units or the LF/HF ratio (as used to compute ANSI) provides the strongest correlation with attendant changes in MSNA particularly if assessed by its amplitude or spectral components rather than in bursts/min” (Pagani et al., 1997). Moreover, the overall number of subjects and the adopted statistical methodology support the hypothesis that a unitary index (Hess, 1949) might indeed furnish a convenient approach to individually assess the *process* (Ahn et al., 2006) of autonomic (dys)regulation. This view also suggests that ANSI could detect sensitively different conditions characterized by impaired autonomic regulation, like coronary artery disease (Sala et al., 2017) and heart failure or even better than normal in professional soccer players (Lucini et al., 2020).

Besides, the study and the statistical methodology we developed consider only one data set. We have tried to overcome this potential limitation using non-parametric bootstrap procedures (Efron and Tibshirani, 1993) to set up confidence intervals and test statistical hypotheses. However, these procedures represent the first step in unanchoring, as much as possible, the analyses to the available data set. A more advanced statistical approach to mimic the presence of more than one data set would require, e.g., a *real* non-parametric bootstrap study, which was not our focus here.

Finally, this is a cross-sectional study. Only longitudinal protocols will possibly overcome this limitation. On the other hand, the relatively large population permits the application of modern statistical tools, capable of unraveling *latent* information, whose clinical value might be evaluated with more rational study protocols.

## Conclusion

The present study suggests, we believe for the first time, a method to assess the strength of the empirical evidence of the information link between obesity and CAR as recapitulated by multiple synthetic statistical indicators extracted from 756 otherwise healthy subjects. These indicators taking under control potential age and gender bias serve as descriptors of the CAR impairment according to a transition process detected toward changes of states due to increasing BMI. In this sense, these indicators are to be treated as process indicators (Ahn et al., 2006), providing a clinically convenient technique to assess cardiovascular autonomic performance individually with simple numerical indicators of rank.

We observe that the pressure domain (through the pressure indicator) shows the highest sensitivity in alterations toward the transition from the normoweight group to the obese group through the overweight group. This finding provides further empirical evidence to the progressive tendency of obesity to foster hypertension independently of age and gender. Moreover, obesity proves also to have significant effects in impairment of the overall ANS state (as described by ANSI (Sala et al., 2017)), cardiac baroreflex regulation (given by the α_*a*__*PRT*_ index, i.e., the age-and-gender-adjusted α index transformed in rank percentiles), and pulse variations (through the pulse indicator). More in detail, the skewed distribution of pressure and pulse indicators, the α_*a*__*PRT*_ index, and ANSI toward the unfavorable extremity of the abscissa particularly evident in the obese group suggests the existence in obese patients of a phenotype characterized by a trend to impaired CAR and increased arterial pressure (Hall et al., 2015). The clinical value of these findings requires further *ad hoc* investigations.

Finally, simplifying the study of autonomic mechanisms may provide further insight into risk stratification and treatment. The growing success of cardiac wearables (Piwek et al., 2016) might, in addition, render short-term HRV within everybody’s reach, likely contributing to a greater appreciation that numerical assessment of autonomic regulation might be conveniently added to the clinical management of overweight and obesity.

## Data Availability Statement

The raw data supporting the conclusions of this article will be made available by the authors, without undue reservation.

## Ethics Statement

The studies involving human participants were reviewed and approved by Independent Ethics Committee of IRCCS Humanitas Clinical Institute (Rozzano, Italy). The patients/participants provided their written informed consent to participate in this study.

## Author Contributions

NS, MP, and DL contributed to the conception and design of the study. NS designed the statistical methodological approach, implemented the R programming codes, and performed the statistical analyses. NS and MP wrote the first draft of the manuscript. NS, MP, and DL contributed to critically revising the text, read, and approved the submitted version.

## Conflict of Interest

The authors declare that the research was conducted in the absence of any commercial or financial relationships that could be construed as a potential conflict of interest.
