# Supplementary material for: Altered Cardiac Autonomic Regulation in Overweight and Obese Subjects: The Role of Age-and-Gender-Adjusted Statistical Indicators of Heart Rate Variability and Cardiac Baroreflex
Source: Front Physiol. 2021 Jan 28;11:567312. doi: 10.3389/fphys.2020.567312 (PMC7876296; doi:10.3389/fphys.2020.567312)
Supplement: Supplementary file 1 [file Table_1.DOCX]

Supplementary Material

**Altered cardiac autonomic regulation in overweight and obese subjects: the role of age-and-gender-adjusted statistical indicators of heart rate variability and cardiac baroreflex**

**Nadia Solaro^1^, Massimo Pagani^2^, Daniela Lucini^2*^ & Fabio Badilini^3^**

^1^Department of Statistics and Quantitative Methods, University of Milano-Bicocca, Milan, Italy

^2^BIOMETRA Department, University of Milan, Milan, Italy

^3^AMPS-LLC, New York, NY, USA, Department of Physiologic Nursing, UCSF, San Francisco, USA

**^*^Correspondence:** Daniela Lucini, MD, PhD: [daniela.lucini@unimi.it](mailto:daniela.lucini@unimi.it)

# Supplementary Methodological Appendix

# Autoregressive Spectral Analysis of RR and Arterial Pressure Variabilities

***Foreword***

In this appendix, we will briefly outline the methodology underlying autoregressive spectral analysis of RR and arterial pressure variabilities, according to our everyday experience with patients or experimental conditions. We will largely reproduce the text published in the original studies and describing methods for AR spectral estimation (Pagani et al., 1986) and the computation of the frequency domain index of cardiac baroreflex gain (Pagani et al., 1988). A study discussing the interpretation of variability of efferent sympathetic nerve activity (Pagani and Malliani, 2000) and a recent update (Lucini et al., 2018) are also suggested for additional details. The software employed to perform the autonomic analysis has been previously described (Badilini et al., 2005) and is available as a commercial tool. An updated overview of the neural structures underlying cardiovascular variabilities is also available (Solaro et al., 2021, submitted).

Addressing the translational approach to studies of the visceral (i.e., autonomic) nervous system must consider the multiple ontologies that come into play: clinical medicine (from prevention Hippocratic style to modern transplant surgery), physiology (normal and pathological), bioengineering (data analysis and modeling computation), epidemiology (ecology and environment), statistics (Gaussian as opposed to distribution-free), modeling (deterministic or stochastic), health-disease (simple causality or complex system dynamics), as well as analog vs. digital, univariate vs. multivariate world.

The risk of misunderstanding and confounding of language is real, sometimes unrecognized, and frequently just incomprehensible. Here we deal with “reciprocal mutual connections between the sympathetic-ergotropic and the parasympathetic-trophotropic areas, at each moment they produce a dynamic equilibrium adapted to the situation at any given moment of the organism as a whole” (Hess, 1949).

As superbly synthetized by RW Hess (Nobel prize in 1949) in spite of multiple organs, individual behavior obeys not to a randomly predominant competitor, but answers to “unitary” dynamic goals of (integrated) individuals. Accordingly, we must deal with descriptions of explicit functions (described in the physical world using absolute measures: mmHg, sec, m, Kg, etc.) and more or less implicit functional control systems (neural, hormonal, immunological, and behavioral), which usually are described using (numerical) metrics dealing with (intangible bits of) information (Kerkhof et al., 2019). In the last decades, this aspect gained explosive momentum thanks to the digitalization of every aspect of daily life.

Following these premises, we start with the seminal idea that “sympathetic and parasympathetic nervous activity make frequency-specific contributions to the heart rate power spectrum” (Akselrod et al., 1981), proposing a strong interaction between physiology and information domains. Accordingly, HR fluctuations could furnish a probe (i.e., proxy) of short-term neural cardiac regulation. A critical task, however, consists in finding ways to make estimates that are both unbiased and in agreement with all possible knowledge (Haken, 1983). A potential confounder derives from the coexistence in the various aspects of nerve signals of both raw quantity (number of informational bits, and eventually mass of synaptic transmitters, e.g., Norepinephrine) and quality (i.e., the dimensionless pattern of discharge, e.g., spectral components or phase angle). These differences cannot be disregarded in the process of interpreting neural information.

Considering that neural control of cardiac functions is instrumental to dynamics of changing requests from the periphery, one might also consider the capacity of HRV (and derived indices) of signaling variations in the autonomic drive (either modeling only peripheral efferent activity or, more correctly the complex hierarchy of the entire control system). Following this integrated (closed-loop, sensory and motor, dynamical) model, unitary indices of HRV gain a more compelling value than the separated vagal or sympathetic (motor only) hypothesis. The dynamics of RR V [RR variability] implies to focus on large, fast changes in raw (e.g., RR variance measured in msec^2^) and computed (e.g., the ratio LF/HF) indices. The combination of both raw and derived variables might, in addition, represent a better approach to cardiovascular metrics helping integrate clinical research with medical care. This last approach was exploited in building a unitary Autonomic Nervous System Index (ANSI) (Sala et al., 2017) that appears particularly useful in translational conditions because of its age and gender independence and percentile rank evaluation. The 0-100 range (higher better) simplifies the appreciation of autonomic performance in individual patients.

***AR Spectral Analysis (Pagani et al., 1986)***

The basic assumption underlying the proposed signal processing methods is that heart rate and systolic and diastolic blood pressure values fluctuate on a cardiac cycle-by-cycle basis, even in stable conditions, around a given mean value. Beat-to-beat heart rate and arterial blood pressure variability signals are derived from the original biological waveform data signals as a discrete time series y(k), where k is the progressive number of the underlying cardiac beat (i.e., the time series sampling clock is the actual heartbeat). These variability signals are intrinsically pseudo-random and can be considered as the realization of a stochastic process y(k), which is the output of a linear time-invariant system driven by a white noise component w(k), i.e., a time series characterized by zero mean and by a variance X^2^ equally distributed over the spectrum of frequencies WN(0, X^2^).

A simple structure of input/output relation of the linear time-invariant system is given by the autoregressive (AR) modelization, which assumes the time series to be the result of a self (auto) regression (the deterministic component) and a white noise component with power X^2^, i.e.:

y(k)=$\sum_{i=1}^{p} a\left( i \right)y(k-i)$ + w(k),

where w(k) is WN(0, X^2^) and where a(i) are the p unknown coefficients (parameters) of the AR model.

Hence, given N samples of variability signals, the problem is to estimate the correct value of p (i.e., the order of the model) and the related p coefficients a(1), a(2), …, a(p) of the (auto)regressing model. This is typically done applying the Levinson-Durbin algorithm, which recursively computes the parameters for increasing values of p, applying minimal least-square prediction error.

Two tests are used to check the validity of the assumed model. The first is Anderson's test, which assesses the whiteness of the prediction error for a given order and rejects the identification if the test is not satisfied within a 5% confidence interval. After fulfilling Anderson's test, the best order of the model is chosen as the one which minimizes Akaike's final prediction error (FPE) figure of merit. In this way, the model is completely determined by order p and by the vector of p estimated parameters.

Power spectral density (PSD) estimation P(f) is obtained by taking the Z-transformation of the model, which in the case of the AR model is totally characterized by the power (variance) of the white noise signal and by the p estimated parameters. The resulting PSD satisfies the criterion of maximum entropy and presents many advantages in respect to the methods employed with classical Fourier analysis (FFT algorithms), namely, a more consistent and smoother spectral estimation, a spectral resolution which is independent of the number N of samples, and the possibility of avoiding data windowing typically employed with FFT methods, which determines a leakage effect in the power spectrum. Another important advantage of AR modeling is the possibility of decomposing the power spectrum in individual components directly associated with the vector of the estimated coefficients, with each of the components being associated with a specific “central” frequency and intensity (power contribution of the component). In this way, the AR-based power spectrum also provides the capability to characterize the individual spectral components, both in terms of their absolute and relative power. (The multiple components from the analysis and their approximate meaning are reported in Table 2 of the main text).

***The multivariate approach and the Alpha index (Baselli et al., 1988)***

AR modeling can be separately applied to the ECG-derived RR interval time series t[k] (or tachogram) and to the arterial pressure systolic time series s[k] (or systogram), leading to the respective auto-spectral analyses of the two signals. By using a multivariate approach, the two synchronous time series can be analyzed with a more complex closed-loop model, which takes into account the influences of one signal, the other one, and vice-versa (Figure MA1).


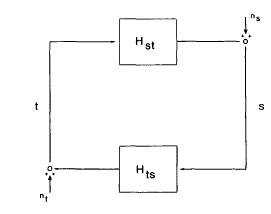


**Methodological Appendix Figure MA1.** Schematic representation of the closed-loop model of the relationship between tachogram (t) and systogram (s). H_ts_ and H_st_ are the transfer functions between the systogram and the tachogram, and vice-versa; n_t_ and n_s_ are two independent exogenous white noise components on the tachogram and the systogram.

The model reported, derived from a control theory approach, cannot be solved without additional a priori information. Therefore, modifying further a suggestion by Akselrod et al. (1985), the relationship between the power density spectra P(f) of input/output signals of the block reported in Figure MA1 is P_t_(f) = |H_ts_(f)|^2^P_s_(f), which can be rewritten as |H_ts_(f)|= (P_t_(f)/P_s_(f))^1/2^ . Namely, the gain of the transfer function between s and t can be computed from the power spectrum of s[k] and t[k]. The gain of H_ts_ is typically computed only at those bands that present a high squared coherence (>0.5), which usually occurs at the LF and HF central frequencies, leading to the so-called α_LF_ and α_HF_ indices.


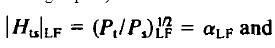


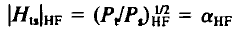


In summary (Pagani et al., 1988), the calculation of α values requires the following steps: 1) calculation of the AR power spectrum and coherence between t[k] and s[k]; 2) automatic determination of LF and HF components through AR spectral decomposition; and 3) calculation of the square root ratio of P_t_(f), and P_s_(f), in two major bands, provided that coherence is larger than 0.5. Usually, we utilize for convenience, like in the present study, the average of the LF and HF gains (as (α_LF_+α_HF_)/2).

***AR Spectral analysis: HeartScope***

As mentioned in the foreword, the analyses performed in our studies were conducted using HeartScope, a software package which was initially developed in the early 90s through a tight collaboration between our research team, namely with Prof. Pagani, Prof Lucini, and Prof. Porta, and US-based software providers with signal processing expertise in the cardiovascular domain (AMPS LLC, New York) (Badilini et al., 2005).

HeartScope is now commercially available (https://www.amps-llc.com/prodotti-holter/software) and has been used by several worldwide research institutes and in the clinical research arena. HeartScope can compute a thorough set of HRV parameters, in both the time and frequency-domains and which include linear (spectral analysis by AR or FFT-based methods), non-linear and symbolic analyses (e.g., conditional entropy), form different cardiovascular biological signals, including muscle sympathetic nervous activity (MSNA).


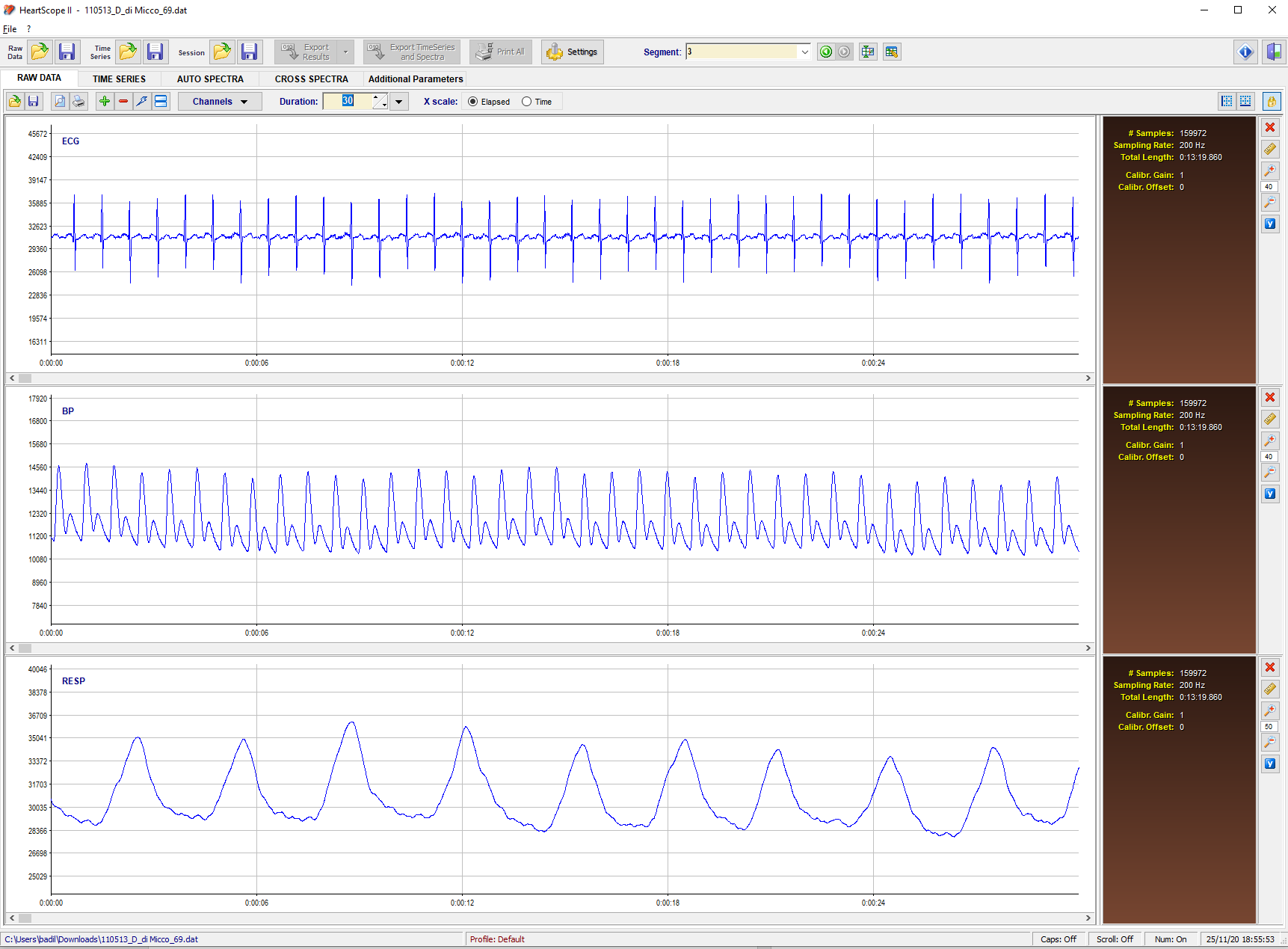


**Methodological Appendix Figure MA2.a.** HeartScope main screen with an example of ECG ABP and respiratory raw waveform signals.


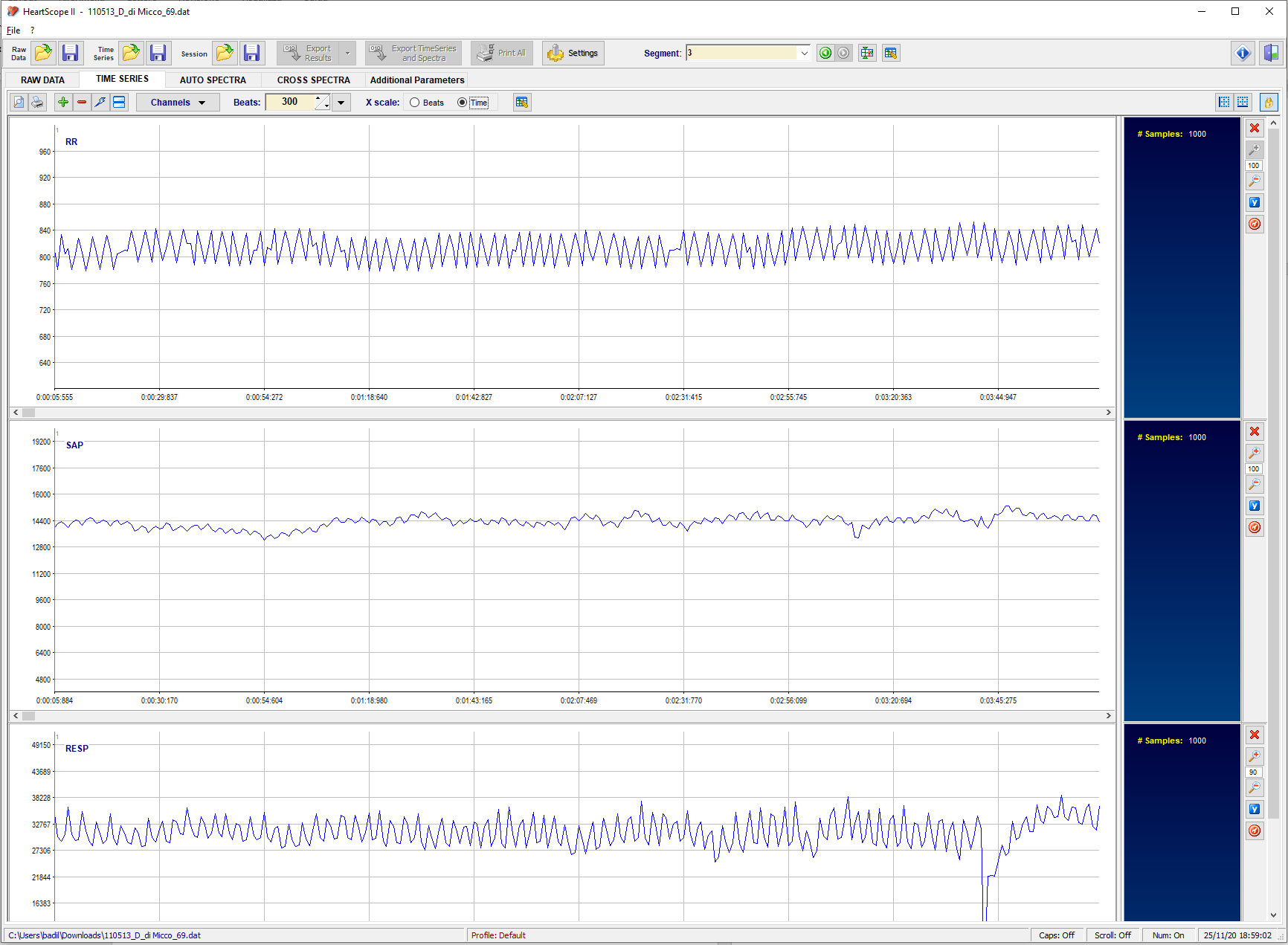


**Methodological Appendix** **Figure MA2.b.** HeartScope time series window derived from waveforms of Figure MA2.a. From top to bottom: RR tachogram t[k], systogram s[k], and respirogram r[k] time series.


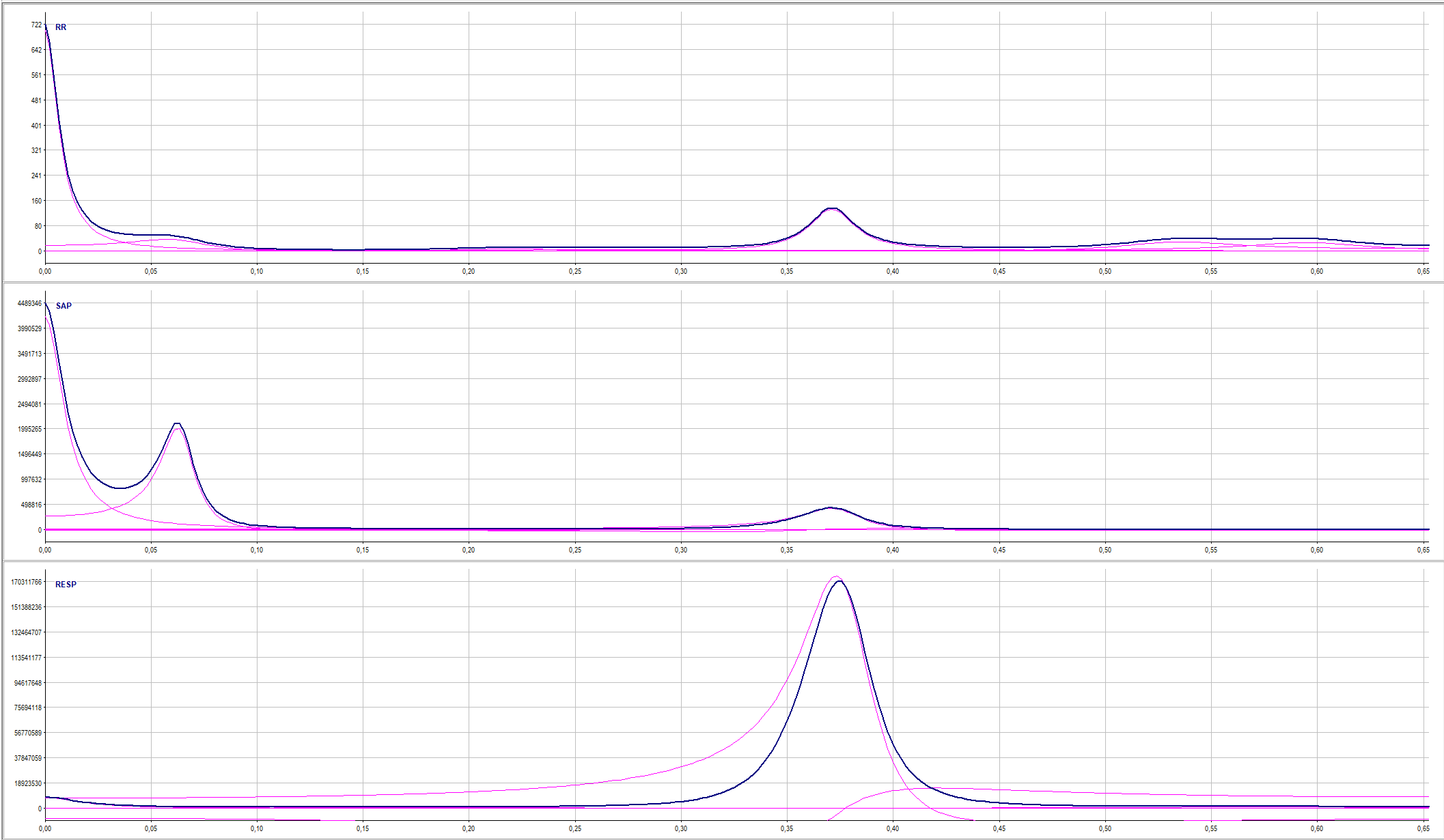

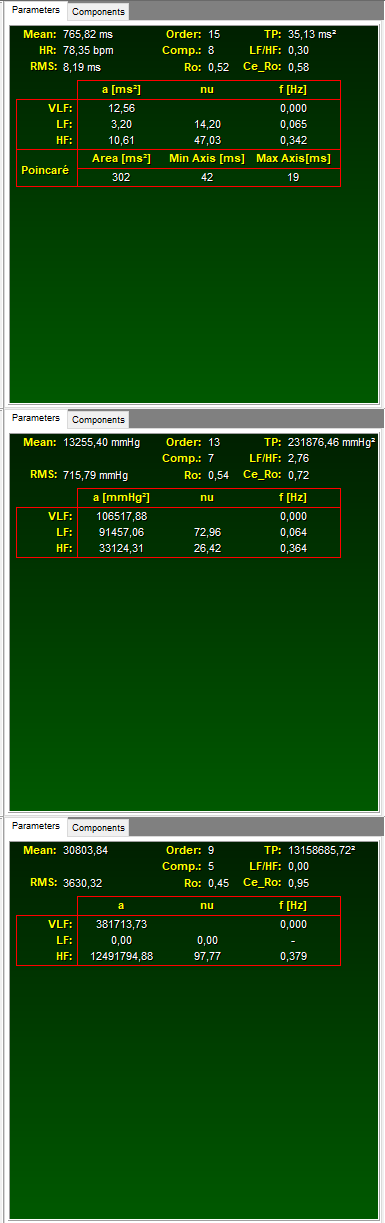


**Methodological Appendix** **Figure MA2.c.** AR power spectra of RR tachogram, systogram, and respirogram from Figure MA2.b. On the right-hand side of each spectrum, the numerical computations.


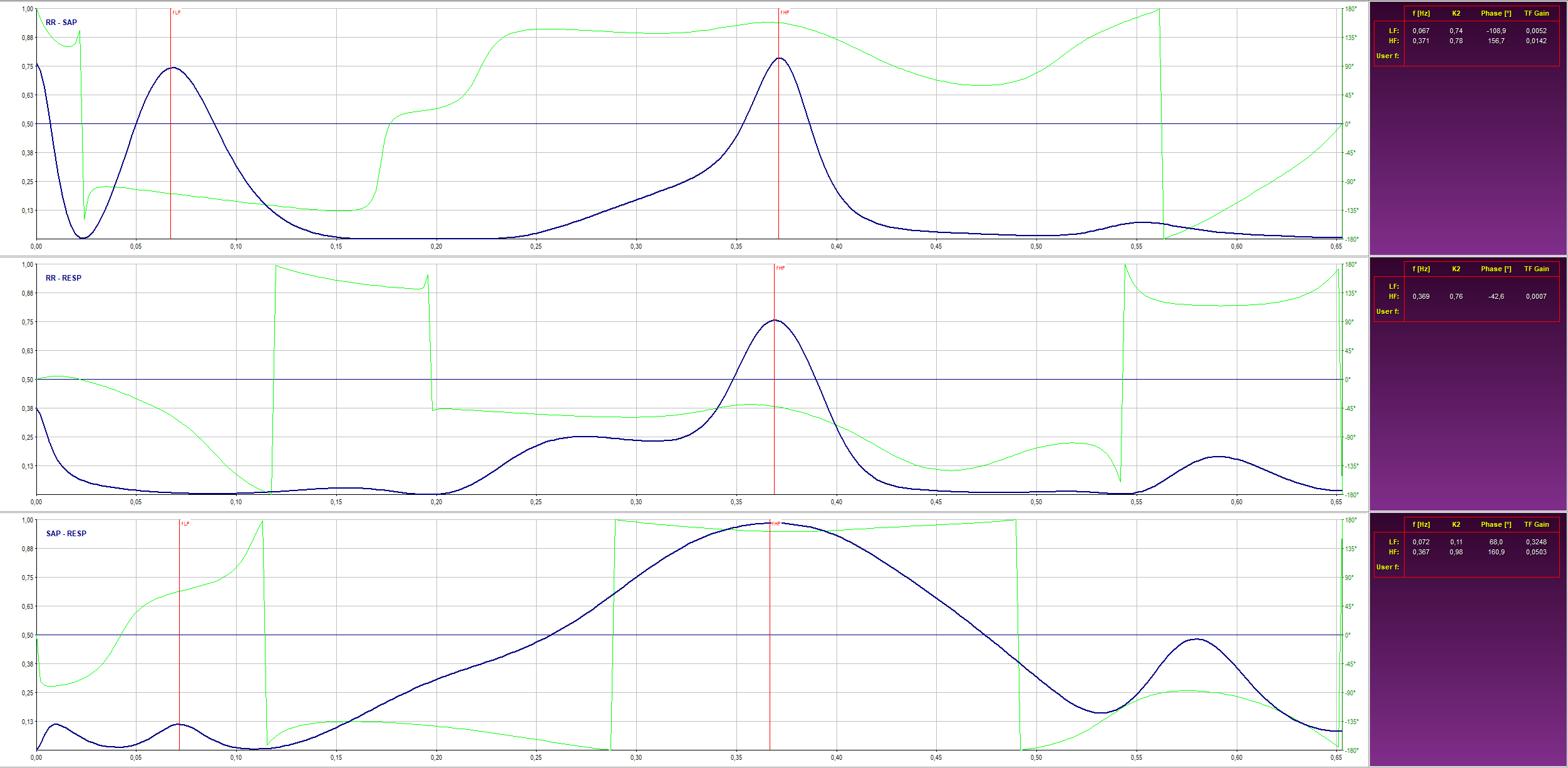


**Methodological Appendix** **Figure MA2.d.** Cross-spectra phase (green line) and squared coherence (blue line) between t[k], s[k] and r[k] time series. On the right-hand side, the numerical results of each cross-spectrum. By looking at the top (RR and SAP), the reported numerical values refer to the central frequency, squared coherence, phase, and gain of the transfer function (the α value), respectively, in the LF and HF bands.

***Conclusions***

Before starting to employ AR spectral analysis to assess cardiovascular neural regulation, a *caveat* is imperative, as there is still an active debate about the usefulness of RR V (or as usually indicated HRV). To answer the radical question posed by J Karemaker many years ago (“Heart rate variability: why do spectral analysis?”, Karemaker, 1997), still more work needs to be done. We will have to wait at least until the semantic issue of autonomic or neurovegetative nervous system is solved.

One wish of HRV seems definitely abandoned: sympathetic and vagal (nerve) activity cannot be measured through RRV. Mono and bivariate spectral analysis of RR and SAP variability signals can, however, provide an indirect assessment of the performance of neural regulation of cardiovascular function. In this context, we are exploring the usefulness of a unitary index ANSI, which seems very promising.

***Suggested references***

Akselrod, S., Gordon, D., Madwed, J. B., Snidman, N. C., Shannon, D. C., Jonathan Cohen, R., et al. (1985). Hemodynamic regulation: investigation by spectral analysis. Am J Physiol. 249, H867-875. doi: 10.1152/ajpheart.1985.249.4.H867

Akselrod, S., Gordon, D., Ubel, F. A., Shannon, D. C., Barger, A. C., and Cohen, R. J. (1981). Power spectrum analysis of heart rate fluctuation: A quantitative probe of beat-to-beat cardiovascular control. Science 213, 220–222. doi: 10.1126/science.6166045

Badilini, F., Pagani, M., and Porta, A. (2005). HeartScope: A software tool addressing autonomic nervous system regulation. Comput Cardiol. 32, 259–262. doi: 10.1109/CIC.2005.1588086

Baselli, G., Cerutti, S., Civardi, S., Malliani, A., and Pagani, M. (1988). Cardiovascular variability signals: towards the identification of a closed-loop model of the neural control mechanisms. IEEE Trans Biomed Eng. 35, 1033–1046. doi: 10.1109/10.8688

Haken, H. (1983). *Synergetics: an introduction*. Berlin: Springer Verlag.

Hess, W. R. (1949). The Central Control of the Activity of Internal Organs. in *Nobel Lecture Physiology or Medicine 1942-1962* (Elsevier Publishing Company).

Karemaker, J. M. (1997). Heart rate variability: Why do spectral analysis? Heart 77, 99–101. doi: 10.1136/hrt.77.2.99

Kerkhof, P. L. M., Peace, R. A., and Handly, N. (2019). Ratiology and a Complementary Class of Metrics for Cardiovascular Investigations. Physiology 34, 250–263. doi: 10.1152/physiol.00056.2018

Lucini, D., Solaro, N., and Pagani, M. (2018). Autonomic differentiation map: A novel statistical tool for interpretation of Heart Rate Variability. Front. Physiol. 9:401. doi: 10.3389/fphys.2018.00401

Pagani, M., Lombardi, F., Guzzetti, S., Rimoldi, O., Furlan, R., Pizzinelli, P., et al. (1986). Power spectral analysis of heart rate and arterial pressure variabilities as a marker of sympatho-vagal interaction in man and conscious dog. Circ. Res. 59, 178–193. doi: 10.1161/01.RES.59.2.178

Pagani, M., and Malliani, A. (2000). Interpreting oscillations of muscle sympathetic nerve activity and heart rate variability. J. Hypertens. 18, 1709–1719. doi: 10.1097/00004872-200018120-00002

Pagani, M., Somers, V., Furlan, R., Dell’Orto, S., Conway, J., Baselli, G., et al. (1988). Changes in autonomic regulation induced by physical training in mild hypertension. Hypertension 12, 600–610. doi: 10.1161/01.HYP.12.6.600

Sala, R., Malacarne, M., Solaro, N., Pagani, M., and Lucini, D. (2017). A composite autonomic index as unitary metric for heart rate variability: a proof of concept. Eur. J. Clin. Invest. 47, 241–249. doi: 10.1111/eci.12730

Solaro, N., Pagani, M., and Lucini, D. (2021). Altered cardiac autonomic regulation in overweight and obese subjects: the role of age-and-gender-adjusted statistical indicators of heart rate variability and cardiac baroreflex. *Main text*

# Supplementary Figures and Tables

## Supplementary Figures

**Supplementary Figure S1.** Box plots of the distributions of the original (odd columns) vs. adjusted (even columns) ANS proxies within the BMI groups.

**
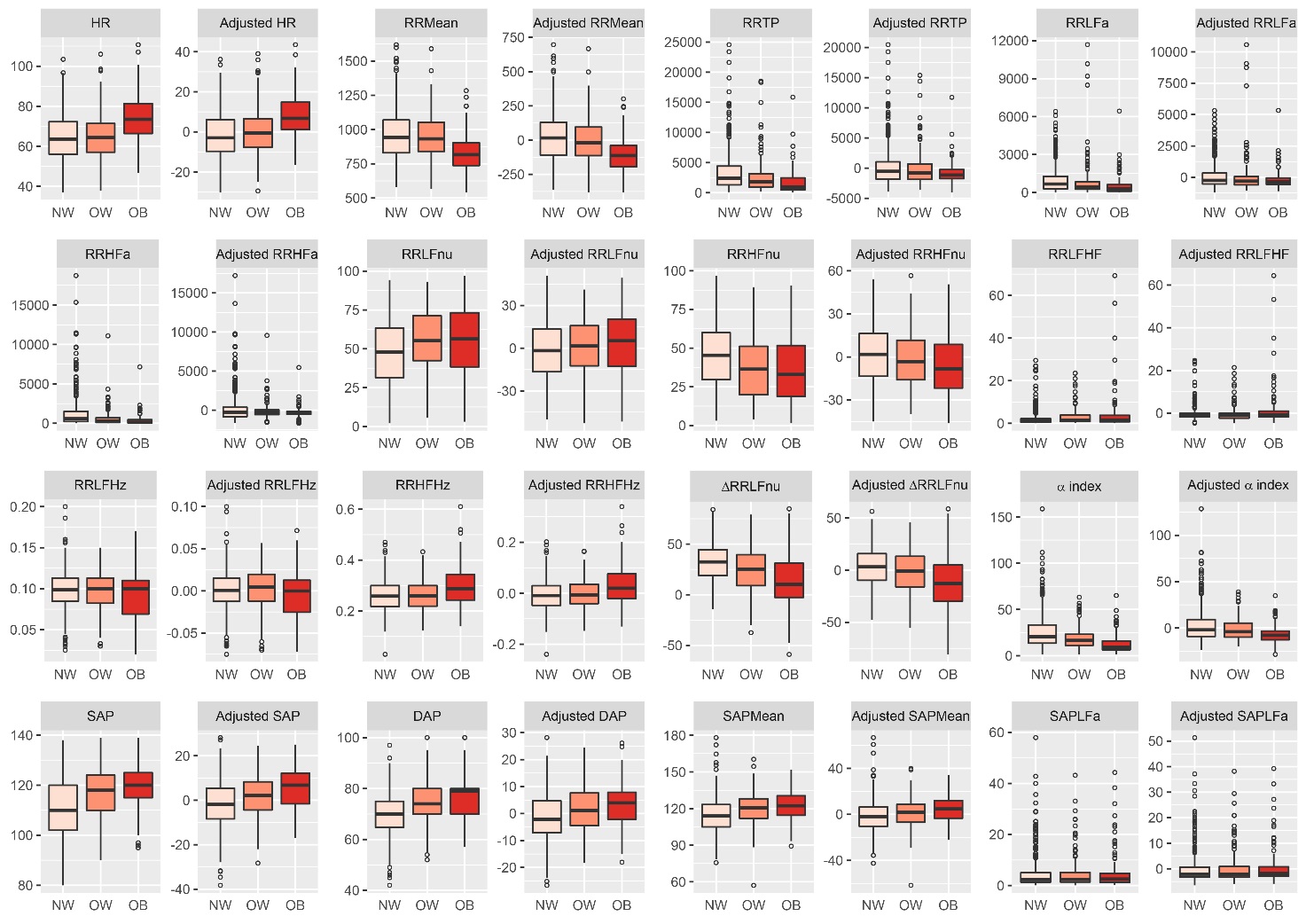
**

*Note.* Adjusted ANS proxies are obtained according to transformation 1 in Figure 1 in the text. The graph is set up with the R software contributed package “ggplot2” (Wickham, 2016).

Figure S1 shows the box plots of the within-BMI-groups distributions of the starting ANS proxies (panels in the odd columns) and the adjusted ANS proxies (panels in the even columns). Overall, two aspects can be noted. First, the boxes of the ANS proxies depicted in each panel, representing the central part of their distribution, evidence specific trends as the groups vary from NW to OB. For example, the boxes of the α index, as well as ΔRR LFnu, tend to have lower ordinates when moving from NW to OB. The opposite trend is observed for the blood pressure measures SAP, DAP, and SAP Mean. Second, the adjusted ANS proxies exhibit, overall, trends very similar to the original ones, e.g., the within-BMI-groups distributions of the adjusted α index and the adjusted ΔRR LFnu are analogous to the corresponding unadjusted proxies. That is a crucial point in that it suggests the presence of meaningful connections of the various ANS states with the BMI conditions even when age and gender effects are taken under control in the analyses.

## Supplementary Tables

**Supplementary Table S1.** Distribution of the participants to the study within the BMI groups by gender and classes of age.

| **Gender** | | | | **BMI groups** | | | *Total* |
| --- | --- | --- | --- | --- | --- | --- | --- |
|  |  |  |  | NW | OW | OB |  |
| Female | **Age in class**  **[yrs]** | 17 – 30 | *count* | 161 | 20 | 18 | 199 |
|  |  |  | *%* | 52.1% | 27.0% | 20.0% | 42.1% |
|  |  | 31 – 49 | *count* | 102 | 38 | 47 | 187 |
|  |  |  | *%* | 33.0% | 51.4% | 52.2% | 39.5% |
|  |  | 50 – 83 | *count* | 46 | 16 | 25 | 87 |
|  |  |  | *%* | 14.9% | 21.6% | 27.8% | 18.4% |
|  | *Total* | | *count* | 309 | 74 | 90 | 473 |
|  |  |  | *%* | 65.3% | 15.6% | 19.0% | 100.0% |
| Male | **Age in class**  **[yrs]** | 17 – 30 | *count* | 96 | 25 | 3 | 124 |
|  |  |  | *%* | 58.9% | 28.1% | 9.7% | 43.8% |
|  |  | 31 – 49 | *count* | 52 | 41 | 17 | 110 |
|  |  |  | *%* | 31.9% | 46.1% | 54.8% | 38.9% |
|  |  | 50 – 83 | *count* | 15 | 23 | 11 | 49 |
|  |  |  | *%* | 9.2% | 25.8% | 35.5% | 17.3% |
|  | *Total* | | *count* | 163 | 89 | 31 | 283 |
|  |  |  | *%* | 57.6% | 31.4% | 11.0% | 100.0% |

*Legend.* Cells in the above 3-way table report:

- in the marginal column, the distribution of absolute counts $n_{hk.}$ and percentages: $p_{k|h}=\frac{n_{hk.}}{n_{h..}}100$ of females ($h=1$, gray part) and male ($h=2$, green part) by classes of age ($k=1,2,3$, with: 1 = 17 – 30 years, 2 = 31 – 49 years, 3 = 50 – 83 years) computed over the BMI groups ($g=1,2,3$, with: 1 = NW, 2 = OW, 3 = OB), where: $n_{h..}=\sum_{k=1}^{3} \sum_{g=1}^{3} n_{hkg}$ and $n_{hk.}=\sum_{g=1}^{3} n_{hkg}$;
- in the marginal (gray and green) rows, the distribution of absolute counts $n_{h.g}$ and percentages: $p_{g|h}=\frac{n_{h.g}}{n_{h..}}100$ of females (gray) and male (green) by BMI groups computed over the three classes of age, where: $n_{h..}=\sum_{k=1}^{3} \sum_{g=1}^{3} n_{hkg}$ and $n_{hk.}=\sum_{g=1}^{3} n_{hkg}$, ($h=1,2$, $k=1,2,3$, and $g=1,2,3$);
- in the (blank) inside part, the absolute counts $n_{hkg}$ of subjects observed in each combination $c_{hkg}$ of gender, classes of age and BMI groups, and the within-BMI-groups percentage distributions of females and males by classes of age, i.e., $p_{k|hg}=\frac{n_{hkg}}{n_{h.g}}100$, ($k=1,2,3$), where $n_{h.g}$ is the number of females or males in the BMI group $g$ computed over the three classes of ages, i.e.: $n_{h.g}=\sum_{k=1}^{3} n_{hkg}$, for all $h,g$.
